# ERK-Induced Activation of TCF Family of SRF Cofactors Initiates a Chromatin Modification Cascade Associated with Transcription

**DOI:** 10.1016/j.molcel.2017.02.005

**Published:** 2017-03-16

**Authors:** Cyril Esnault, Francesco Gualdrini, Stuart Horswell, Gavin Kelly, Aengus Stewart, Phil East, Nik Matthews, Richard Treisman

**Affiliations:** 1 Signalling and Transcription Group, Francis Crick Institute, 1 Midland Road, London NW1 1AT, UK; 2 Bioinformatics and Biostatistics STP, Francis Crick Institute, 1 Midland Road, London NW1 1AT, UK; 3 Advanced Sequencing STP, Francis Crick Institute, 1 Midland Road, London NW1 1AT, UK

**Keywords:** SRF, Elk-1, ternary complex factor, H3 phosphorylation, histone modification, immediate-early genes, transcription, chromatin, ERK

## Abstract

We investigated the relationship among ERK signaling, histone modifications, and transcription factor activity, focusing on the ERK-regulated ternary complex factor family of SRF partner proteins. In MEFs, activation of ERK by TPA stimulation induced a common pattern of H3K9acS10ph, H4K16ac, H3K27ac, H3K9acK14ac, and H3K4me3 at hundreds of transcription start site (TSS) regions and remote regulatory sites. The magnitude of the increase in histone modification correlated well with changes in transcription. H3K9acS10ph preceded the other modifications. Most induced changes were TCF dependent, but TCF-independent TSSs exhibited the same hierarchy, indicating that it reflects gene activation per se. Studies with TCF Elk-1 mutants showed that TCF-dependent ERK-induced histone modifications required Elk-1 to be phosphorylated and competent to activate transcription. Analysis of direct TCF-SRF target genes and chromatin modifiers confirmed this and showed that H3S10ph required only Elk-1 phosphorylation. Induction of histone modifications following ERK stimulation is thus directed by transcription factor activation and transcription.

## Highlights

• TPA-driven ERK activation induces a common pattern of histone modifications at TSSs
• Most ERK-induced histone modifications are dependent on TCF family of SRF cofactors
• At these TSSs, induced histone modifications require ERK-dependent TCF phosphorylation
• TCF-dependent histone modifications require TCF to recruit the transcription machinery

## Graphical Abstract

**Figure:**
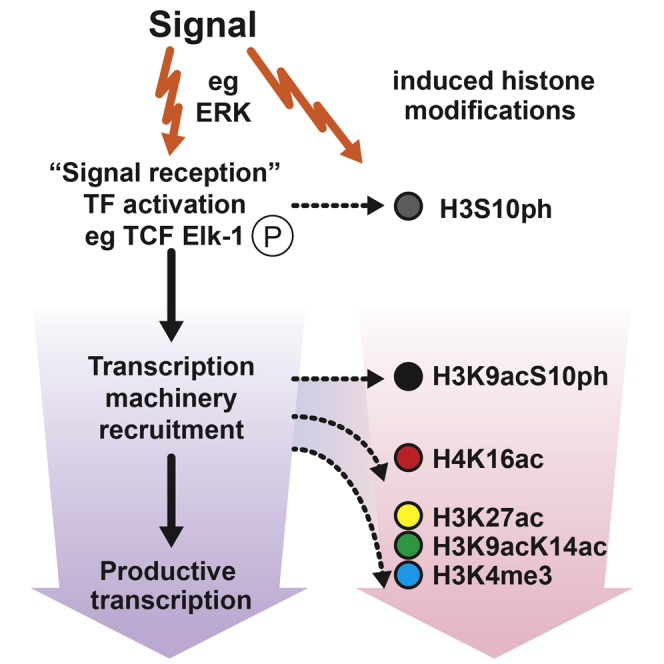

## Teaser

In MEFs, TPA-induced ERK activation induces a common pattern of histone modifications at more than 2,000 TSSs. Modifications at most TSSs are dependent on the TCF family of ERK-regulated SRF cofactors. At these TSSs, induced modifications are dependent both on phosphorylation of TCF and on its ability to recruit the transcriptional machinery.

## Introduction

Specific patterns of chromatin modification correlate with gene activation or silencing and can be used to identify gene regulatory elements such as promoters and enhancers (Ernst and Kellis, 2010, Ernst et al., 2011, Hon et al., 2009, Koch et al., 2007, Li et al., 2007, Wang et al., 2008). Modifications associated with transcriptionally active genes include H3K4me3 (Liang et al., 2004, Ng et al., 2003, Pokholok et al., 2005, Santos-Rosa et al., 2002, Schneider et al., 2004), H3K9acK14ac (Bernstein et al., 2005, Liang et al., 2004, Pokholok et al., 2005), H3K27ac (Tie et al., 2009, Wang et al., 2008), and H4K16ac (Gelbart et al., 2009, Zippo et al., 2009). It is not clear whether “activating” histone modifications facilitate transcriptional activation or arise independently (Henikoff and Shilatifard, 2011, Lee et al., 2010), although recent advances in transcriptional imaging and gene targeting are now providing insights (Balamotis et al., 2009, Lau and Cheung, 2011, Stasevich et al., 2014, Thakore et al., 2016).

Phosphorylation of H3 at serine 10 and 28, first seen to be associated with mitogen- and stress-induced gene activation (Clayton et al., 2000; for review, see Healy et al., 2012, Mahadevan et al., 1991, Soloaga et al., 2003, Zhong et al., 2001), accompanies gene activation induced by NFκB, HSF, progesterone receptor, and ectopic Gal4 expression (Anest et al., 2003, Labrador and Corces, 2003, Nowak and Corces, 2000, Vicent et al., 2006, Yamamoto et al., 2003). In different contexts, H3 phosphorylation has been associated with recruitment of regulatory cofactors, chromatin modifiers, and remodelers (Cheung et al., 2000, Drobic et al., 2010, Josefowicz et al., 2016, Lo et al., 2000, Zippo et al., 2009), and with displacement of repressors (Lau and Cheung, 2011, Sawicka et al., 2014). For example, at mitogen-induced immediate-early genes, initial H3 phosphorylation is followed by nucleosome remodeling and appearance of H3K9ac and H3K4me3 as transcription increases (Drobic et al., 2010, Riffo-Campos et al., 2015, Zippo et al., 2007, Zippo et al., 2009). Nevertheless, the role played by specific transcription factors in the induction of chromatin modifications such as H3 phosphorylation, and their relationship to transcriptional activation, has remained unclear.

The transcription factor SRF (serum response factor), which controls many immediate-early genes, provides a system to investigate this issue. SRF associates with two families of signal-regulated cofactors, the ERK-regulated ternary complex factors (TCFs) Elk-1, SAP-1, and NET (ELK1, ELK4, and ELK3 in mouse), and the Rho-actin-controlled Myocardin-related transcription factors MRTF-A and MRTF-B (MKL1 and MKL2 in mouse) to regulate proliferation, cell growth, and cytoskeletal dynamics (Buchwalter et al., 2004, Olson and Nordheim, 2010). Stimulation with 12-O-tetradecanoyl phorbol-13-acetate (TPA), which activates ERK, but not Rho-actin signaling (Griner and Kazanietz, 2007), activates almost 3,500 genes, including more than 700 direct SRF genomic targets, in a largely TCF-dependent fashion (Gualdrini et al., 2016). In contrast, the myocardin-related transcription factors (MRTFs) play a major role in the response to serum mitogens, which activate both Rho and ERK, activating many immediate-early and cytoskeletal genes (Esnault et al., 2014). The TCF and MRTF families act antagonistically, their balance affecting cell proliferation and adhesive properties (Gualdrini et al., 2016).

Here we investigated the role of the TCFs in the establishment of ERK-regulated histone modifications at transcription start sites (TSSs), which were identified without prior reference to transcriptional status. TPA stimulation induced an ordered cascade of modifications at these TSSs, whose magnitude correlated well with the increase in transcription. Signal-induced changes at most TSSs were TCF dependent, but similar patterns occurred both at direct TCF-SRF genomic targets and TCF-independent TSSs. Induction of TCF-dependent histone modifications required phosphorylation of the TCF activation domain and its competence to recruit the transcriptional machinery. Analysis of candidate modifiers required for the activation of direct TCF-SRF target TSSs showed that phosphorylation of H3S10 is an early transcription-independent step in the activation process.

## Results

### TPA Stimulation Induces a Common Pattern of Histone Modifications in TSS Regions

We sought to identify chromatin changes induced by ERK signaling in mouse embryonic fibroblasts (MEFs) in an unbiased manner, without pre-selecting sites for analysis according to transcriptional activity. Histone modifications at RefSeq TSS regions were quantified genome-wide by chromatin immunoprecipitation sequencing (ChIP-seq) in resting and TPA-stimulated cells, and those TSSs exhibiting significant change were identified using DESeq (Anders and Huber, 2010) (Table S1). The analysis used antibodies that detect modifications associated with transcriptional activation, including H3K9acS10ph (Mahadevan et al., 1991, Vicent et al., 2006), H4K16ac (Zippo et al., 2009), H3K27ac (Wang et al., 2008), and H3K9acK14ac and H3K4me3 (Guenther et al., 2007). The H3K9acS10ph antibody we used cross-reacts with H3K27acS28ph (Rothbart et al., 2015), and therefore the H3K9acS10ph data presented below potentially represent both epitopes (see Discussion). We note that the H3K27Ac antibody cross-reacts with H3 acetylated at other lysines (Rothbart et al., 2015), but its profile is distinct from H3K9acK14ac (see STAR Methods and Figure S1).

**Figure 1:**
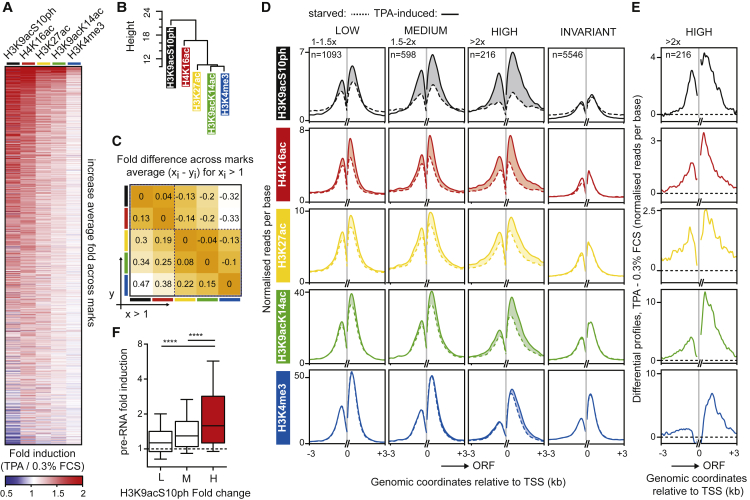
Hierarchy of TPA-Induced Histone Modifications at TSS Regions in MEFs Histone modifications at annotated TSSs were assessed by ChIP-seq using antibodies H3K9acS10ph, H4K16ac; H3K27ac, H3K9acK14ac, and H3K4me3. (A) The heatmap shows changes in ChIP-seq signal across at 2,364 TSS regions (−2 to +1 kb), where at least one modification shows a statistically significant change according to DESeq (among ∼12,000 with detectable ChIP-seq signal). Ranking is by average fold change. (B) Dendrogram plot for the five histone modifications (distance method “euclidean,” nesting method “single”). (C) Average difference in fold induction per compared pair of histone modifications (average of x_i_ − y_i_, for x_i_ > 1). (D) Metaprofiles for the 2,364 TSS regions, grouped by change in H3K9acS10ph (low, 1–1.5× [n = 1,093]; medium, 1.5–2× [n = 598]; high, ≥2× [n = 216]) and TSS regions exhibiting unchanged histone modifications (≥100 reads across each TSS; n = 5,546). ChIP-seq read counts extend 3 kb either side of the TSS. 150 bp on each side of the TSS, where low read counts presumably reflect nucleosome depletion, were excluded. Shaded areas denote difference between the TPA-induced and uninduced levels. (E) Differential profiles (TPA −0.3% FCS) per base at TSS regions displaying >2× increased H3K9acS10ph. For other TSS region classes, see Figure S2C. (F) Boxplot representation of average fold induction of precursor RNA (intronic RNA-seq reads) of the TSS-associated genes as a function of H3K9acS10ph fold change (middle line, median; top and bottom edges, 75th and 25th percentiles). For data summary, see Tables S1 and S2.

TPA stimulation activated ERK within 5 min, and transcription of TCF direct target genes peaked at 15–30 min (Gualdrini et al., 2016), so we examined changes at 30 min to avoid confusion with changes arising from downregulation. A total of 2,364 TSS regions exhibited statistically significant changes in at least one modification (Figures 1A and S1; Table S2A) (see GEO: GSE75002). Hierarchical cluster analysis of the five marks suggested that changes in the different marks exhibit a nested relationship, placing them in the order H3K9acS10ph, H4K16ac, H3K27ac, H3K9acK14ac, and H3K4me3, with two clear subsets comprising the first two and the latter three (Figure 1B). Multiscale bootstrap resampling demonstrated that the changes in the different modifications at different TSSs fall on a continuum rather than representing multiple qualitatively different patterns (Figure S2A).

We calculated a distance index (DI), defined as the average difference between the change in a given modification (the “leading mark”) and that observed for any other mark (see Supplemental Information). Considering H3K9acS10ph as the leading mark, the least dissimilar modification is H4K16ac (13% difference), followed by H3K27ac (30%), H3K9acK14ac (34%), and H3K4me3 (47%) (Figure 1C); this relative order of similarity is maintained when the other modifications are considered as the leading mark (Figure S2B). Thus, the five histone modifications can be placed in the order H3K9acS10ph, H4K16ac, H3K27ac, H3K9acK14ac, and H3K4me3 according to the magnitude of the TPA-induced change at each TSS. Several studies have implicated H3 phosphorylation as an early step in the response to ERK and SAPK activation (for review, see Healy et al., 2012), consistent with the notion that this ordering might reflect a temporal sequence (see below).

### Histone Modifications Correlate with Transcriptional Induction

Having used an unbiased approach to identify a set of TSSs that exhibit signal-induced changes in histone modifications, we next studied the relation between signal-induced histone changes and transcriptional activation. For this, we used an RNA sequencing (RNA-seq) data set measuring changes in RNA synthesis conducted in parallel with the histone modification analysis (GEO: GSE75667) (Gualdrini et al., 2016). First, we divided the TSSs into three classes according to the magnitude of the induced change in H3K9acS10ph (low, 1–1.5×; medium, 1.5–2.0×; high, >2×). Metaprofiles of each mark (Figure 1D) showed that H3K9acS10ph exhibited the largest proportional change upon stimulation and H3K4me3 the smallest; the changes in H3K4me3, and to a lesser extent H3K9acK14ac, were biased in the direction of productive transcription (Figures 1E and S2C). The presence of H3K4me3 in transcription units has previously been correlated with active transcription (Benayoun et al., 2014, Chen et al., 2015, Guenther et al., 2007). As the TPA-induced change in each histone mark increased, so did the increase in TPA-induced intronic RNA-seq reads, diagnostic of increased transcription (Figure 1F).

To gain more quantitative insight into the relation between changes in histone modifications and transcription, we used linear regression analysis. TPA-induced changes in histone modification status at each TSS correlated significantly with the change in RNA synthesis from its associated gene, with R^2^ values ranging from 0.07 (H3K9acS10ph) to 0.28 (H3K4me3) (Figure S3A). We also investigated multiple regression models using a stepwise model selection approach, which includes only variables that allow significant improvement to the model. The most satisfactory was an interdependent model involving all five histone modifications, with an adjusted R^2^ value of 0.4 (p < 2.2 × 10^−16^) (Figures S3B and S3C). Taken together, these experiments show that signal-induced changes in histone modifications at TSSs correlate well with degree of transcriptional induction of their associated genes.

### Most TPA-Induced Chromatin Changes Require the TCFs

**Figure 2:**
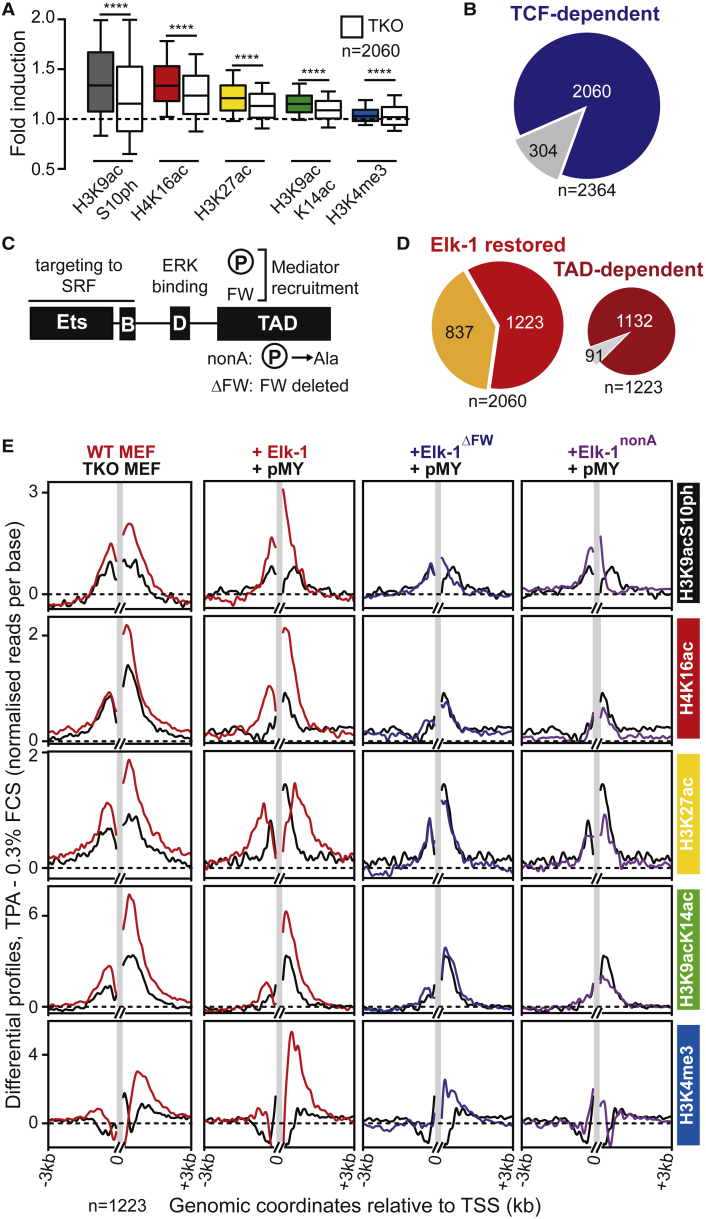
Most TPA-Induced Histone Modifications Are TCF Dependent Histone modifications at annotated TSSs were assessed in wild-type and TKO MEFs as in Figure 1. (A) Comparison of ChIP-seq signal in wild-type and TKO MEFs (colored and white boxes, respectively) at the 2,060 TCF-dependent TSS regions. Middle line, median; top and bottom edges, 75th and 25th percentiles; horizontal bars, 90th and 10th percentiles. Statistical significance by Wilcoxon matched-pairs signed rank test (^∗∗∗∗^p < 0.0001). (B) TCF dependence of TPA-induced changes in histone modifications. (C) Domain structure of Elk-1 (Buchwalter et al., 2004): in Elk-1^nonA^, all ERK sites in the activation domain (TAD) are substituted by alanine; Elk-1^ΔFW^ lacks the FW motif required for Mediator recruitment (Balamotis et al., 2009). (D) Proportion of TSS regions where expression of wild-type Elk-1 restored regulated histone modifications in TKO MEFs. (E) Differential profiles, as in Figure 1E, of ChIP-seq signals across the 1,248 TSS regions where regulated histone modifications is restored by Elk-1. Left: profiles in wild-type (red) and TKO MEFs (black); right: differential profiles in TKO MEFs reconstituted with wild-type Elk-1 (red), Elk-1^ΔFW^ (blue), Elk-1^nonA^ (purple), or pMY vector (black) (Gualdrini et al., 2016). See Figure S4 for full data.

To investigate how the TCFs contribute to the TPA-induced chromatin response, we analyzed histone modifications in MEFs that lack all three proteins (*Elk1*^−/−^, *Elk3*^δ/δ^, and *Elk4*^−/−^ TKO [triple-knockout] MEFs) (Costello et al., 2010). A similar analysis of the immediate transcriptional response using RNA-seq and ChIP-seq found that it is predominantly TCF dependent (Gualdrini et al., 2016). TPA-induced histone modification at classical TCF-SRF target genes was substantially impaired in TKO MEFs (Figure S1), and metaprofiling across the 2,364 TSS regions displaying TPA-induced changes in wild-type MEFs showed that induction of each of the five marks was impaired in TKO MEFs (Figure S4A). We next estimated the number of TSS regions at which the TPA-induced increase in histone modifications was TCF dependent. For each modification, we compared the difference between fold change induced by TPA in wild-type and TKO MEFs with the fold change in wild-type cells. In this analysis, systematic impairment of induction in the TCF KO genetic background should generate a distribution asymmetrically disposed about y = 0, and linear regression analysis should yield slopes that are significantly positive. In contrast, differences arising from random technical variations will be symmetric around y = 0, with a linear regression slope of zero. Regression analysis indicated that all five marks were strongly and positively dependent on TCF activity, with H3K9acS10ph showing the strongest dependence (slope = 0.9, Spearman’s r = 0.7; Figure S4B, top). Moreover, more than 80% (2,060 of 2,364) of TSS regions showed at least one TCF-dependent change in histone modification (Figures 2A, 2B, and S4C).

To investigate the relationship among ERK signaling, TCF activation, and histone modifications, TKO MEFs were reconstituted with retroviruses expressing wild-type human Elk-1 or its inactive derivative Elk-1^nonA^, which cannot be phosphorylated, or Elk-1^ΔFW^, which can be phosphorylated but cannot recruit Mediator (Balamotis et al., 2009) (Figure 2C). All the derivatives generated similar ChIP-seq profiles, indicating that Elk-1 recruitment to SRF occurs independently of phosphorylation (Gualdrini et al., 2016; see also Figure S5C). Re-expression of wild-type Elk-1 was sufficient to restore TPA-induced chromatin changes at ∼60% of TCF-dependent TSS regions (1,223 of 2,060) (Figures 2D, 2E, S4B, S4D, and S4E; Table S2), and at the vast majority of these (1,132 of 1,223) induced histone modifications required Elk-1 phosphorylation and its ability to recruit Mediator (Figures 2D, 2E, S4B, S4D, and S4E; Table S2A). Taken together, these results show that the majority of TPA-induced changes in histone modifications at TSSs are TCF dependent and require ERK-induced transcriptional activation by the TCFs.

### The Response of TCF-Dependent and TCF-Independent TSSs to TPA Is Similar

**Figure 3:**
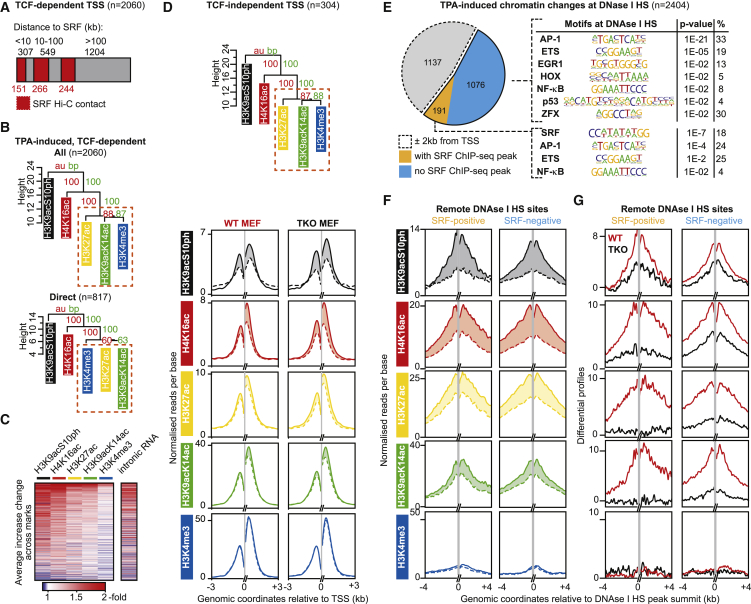
Signal-Induced Histone Modifications at TSSs Are Not Transcription Factor or Promoter Specific (A) Definition of SRF-linked TSSs exhibiting TCF-dependent TPA-induced histone modifications. TSSs are classified by the closest SRF ChIP-seq peak, with TSSs displaying Hi-C interaction with SRF shaded in red. “Direct” TCF-SRF controlled TSSs (n = 817) are defined as all those within 10 kb of an SRF site or that interact in Hi-C. (B) Hierarchy of TPA-induced histone modification changes at all TCF-dependent TSS regions (top) and direct TCF-dependent TSSs (bottom) displayed as in Figure 1B (see also Figure S2A). (C). Heatmap representation of the ChIP-seq signals at the 817 direct TCF-SRF controlled TSS regions, ranked by average fold change, compared with TPA-induced change in RNA synthesis. (D) Hierarchy of TPA-induced histone modification changes at the 304 TCF-independent TSS regions, with metaprofiles of the modifications, plotted as in Figure 1D, shown below. (E) Left: classification of the 2,404 DNase I HS sites showing significant changes in ChIP-seq signals. Gray, sites within 2 kb of a TSS; orange, remote sites with SRF ChIP-seq peaks; blue, others. Sequence motifs enriched at remote DNase I HS are shown at the right. (F) Differential metaprofiles of induced histone modifications at remote DNase I HS sites in wild-type MEFs; shading indicates change upon induction. (G) Differential metaprofiles of histone modification changes at the remote DNase I HS sites in wild-type (red line) and TKO (black lines) MEFs.

We next investigated to what extent the pattern of TPA-induced changes in histone modification was specific to direct gene activation via the TCF-SRF pathway. TSSs exhibiting TCF-dependent induced chromatin changes will include both direct targets of the TCF-SRF complex and indirect targets whose activation is dependent on the products of TCF-controlled genes. To identify direct targets for TCF-SRF-induced histone modification in an unbiased manner, we combined our ChIP-seq analysis of SRF binding in wild-type and TKO MEFs with recent Hi-C analyses of chromosomal contacts in MEFs (GEO: GSE75667) (Gualdrini et al., 2016). This approach identified 817 TSSs that exhibit TCF-dependent histone modifications and are either in close proximity to an SRF site or physically linked to one, as assessed by Hi-C (direct TCF-SRF targets; Figure 3A; Table S2A). The induced modifications at these direct TCF-SRF target TSSs displayed effectively the same hierarchy as that of the TCF-dependent dataset as a whole (Figure 3B). Again, the fold changes in histone modifications at the direct targets correlated well with RNA production (adjusted R^2^ = 0.5, p < 2.2 × 10^−16^) (Figures 3C and S3D). Strikingly, the TCF-independent TSSs displayed an identical hierarchy to that seen at TCF-dependent TSSs (Figure 3D). Taken together, these data suggest that the hierarchy of TPA-induced changes in histone modifications reflects a process underlying gene activation per se rather than a specific pattern associated with gene activation by TCF-SRF signaling.

### Signal-Induced Modifications at Remote Regulatory Sites

Signal-induced chromatin modification at sites remote from TSSs is also associated with enhancer activation and enhancer RNA (eRNA) synthesis (Kim et al., 2010, Lam et al., 2014), so we also examined histone modifications at DNase I hypersensitive (DNase I HS) sites, which are reliable indicators of transcription factor binding (Thurman et al., 2012). Using the approach presented above, we identified TPA-induced changes at sequences previously defined as DNase I HS in mouse fibroblasts (see STAR Methods). Of ∼50,000 DNase I HS sites passing the signal threshold for analysis, 2,404 displayed statistically significant TPA-induced changes in any one of the five marks examined (Table S2B). Of these, 1,267 were >2 kb from TSSs and thus represent potential remote regulatory sites, but only 15% coincided with SRF ChIP-seq peaks (Figure 3E; Table S2B). HOMER motif analysis indicated that these “inducible” DNase I HS sites were significantly enriched in sequence motifs associated with IE transcription factors, including AP-1, Ets, and Egr families, compared with the DNase I HS population as a whole (Figure 3E). At these remote regulatory sites, TPA induced all five histone modifications examined, although H3K4me3 was much less pronounced than the others (Figure 3F). Signal-induced histone modifications at both classes of remote DNase I HS were substantially TCF dependent (Figure 3G).

### H3S10 Phosphorylation Is an Early and General Feature of Transcriptional Activation

**Figure 4:**
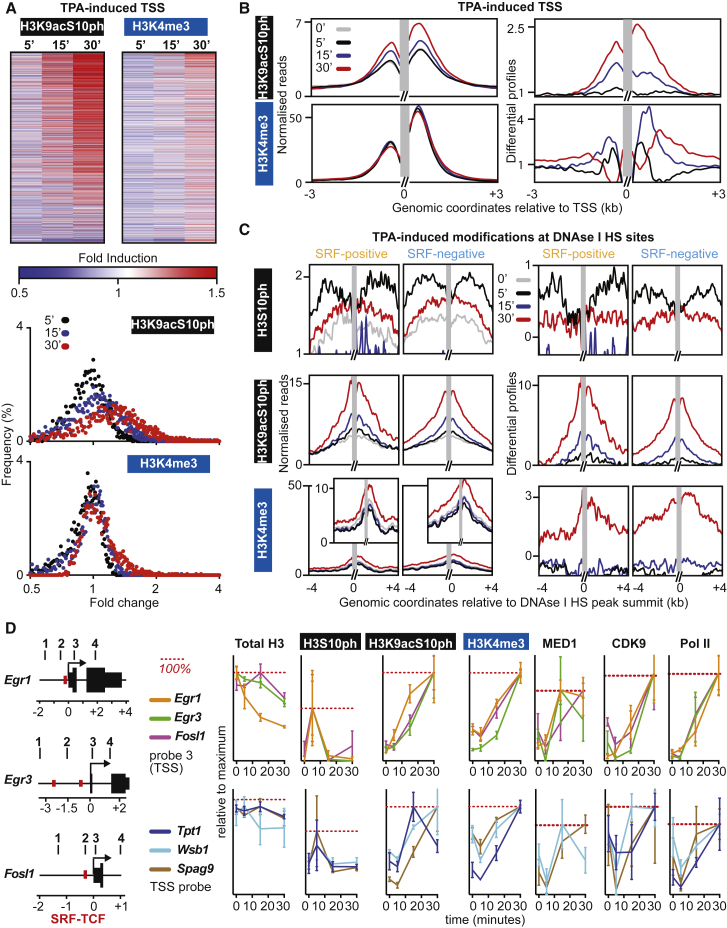
Ordered Histone Modification and Transcriptional Initiation Events at TPA-Induced Genes (A) Top: time course of H3K9acS10ph and H3K4me3 antibody ChIP-seq signal at TPA-inducible TSSs, ranked as in Figure 1A. Bottom: frequency distribution of fold change. (B) Time course metaprofiles (gray, resting; black, 5 min; blue, 15 min; red, 30 min) and differential profiles of H3K9acS10ph and H3K4me3 signal at TSSs where H3K9acS10ph is increased by 30 min TPA stimulation (n = 1,907). (C) Time course metaprofiles and differential profiles of H3S10ph, H3K9acS10ph, and H3K4me3 at remote SRF-positive and SRF-negative DNase I HS sites from Figure 3E, displayed as in (A). (D) Left: TCF direct target genes *Egr1*, *Egr3*, and *Fosl1*; red bars, SRF/TCF binding sites; black bars, PCR probe locations. Right: quantitative ChIP time courses of total H3, H3S10ph, H3K9acS10ph, H3K4me3, MED1, CDK9, and PolII normalized to the maximum detected signal (red dotted line) at the TSSs of TCF targets *Egr1*, *Egr3*, and *Fosl1* and the TCF-independent TPA-induced TSSs *Tpt1*, *Wsb1*, and *Spag9*. Data are mean ± SEM; n = 4. See Figures S5A and S5B.

One potential explanation for the nested hierarchy of changes in histone modification upon TPA stimulation observed above is that it at least in part reflects differences in the temporal order of their establishment. We analyzed the time course of TPA-induced H3 phosphoacetylation and H3K4 trimethylation, across the whole TSS population. In addition, we examined H3S10 phosphorylation directly, using an H3S10ph-specific antibody whose cognate epitope is occluded by H3K9 acetylation (Rothbart et al., 2015). Although a substantial fraction of TSSs exhibited increased H3 phosphoacetylation at 15 min following stimulation, H3K4me3 modification was unchanged at this time, with a subset of TSSs exhibiting substantial increase by 30 min (Figure 4A). Consistent with this, metaprofile analysis revealed that although the H3 phosphoacetylation profile increased uniformly with time, the H3K4me3 modification profile spread within transcribed sequences at later times (Figure 4B). Low read counts precluded de novo analysis of H3S10ph at individual TSSs, but we were able to use metaprofile analysis to study this modification at the TPA-inducible putative remote regulatory DNase I HS identified in the preceding section. Here, increased H3S10ph was detectable at 5 min following stimulation and was replaced by H3K9acS10ph at 15 min, reflecting occlusion of the H3S10ph epitope; H3K4me3 was detectable only at 30 min, at a low level (Figure 4C).

Similar results were observed when we used quantitative ChIP to analyze TPA-induced histone modifications at specific TPA-inducible genes (Figure 4D) (Gualdrini et al., 2016). At both direct TCF-SRF target genes (*Egr1*, *Egr3*, and *Fosl1*) and TCF-independent inducible genes (*Wsb1*, *Tpt1*, and *Spag9*), phosphorylation of H3S10ph was readily detectable at 5 min, preceding the other modifications, and well before the onset of Mediator, Cdk9, and PolII recruitment (Figures 4D, S5A, and S5B). Taken together, these data support a model in which H3S10 phosphorylation precedes the establishment of the other modifications, and the later spread of H3K4me3 reflects induced transcription.

### Induced Histone Modifications at Direct TCF-SRF Targets Require Elk-1 Phosphorylation

**Figure 5:**
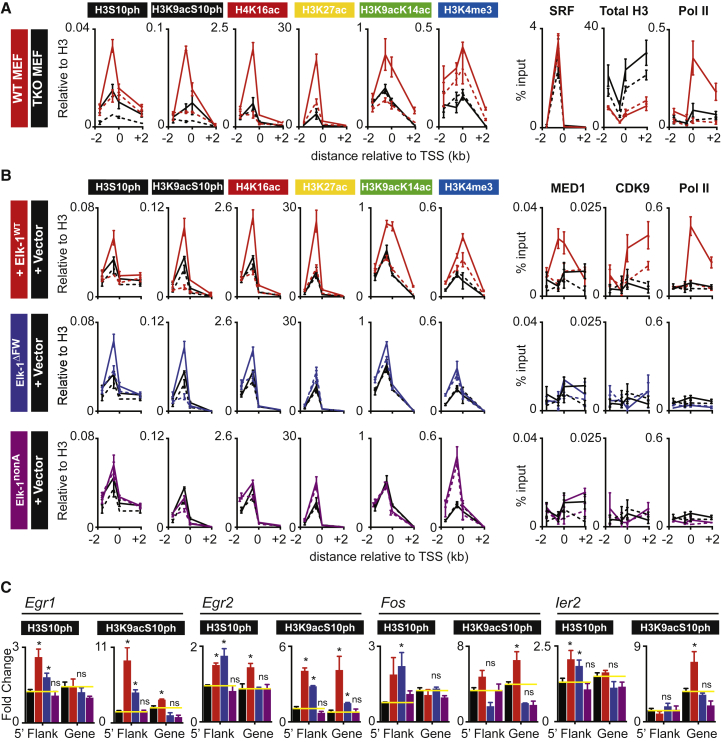
Function of the Elk-1 Transcriptional Activation Domain in Signal-Induced Histone Modifications at *Egr1* (A) Quantitative ChIP at *Egr1* with the indicated antibodies. Dashed lines, unstimulated cells; solid lines, TPA-stimulated cells; red, wild-type MEFs; black, TKO MEFs. Histone ChIP signals are normalized to total H3 (see Figure S5C). (B) Quantitative ChIP analysis of histone modification and transcriptional machinery recruitment at *Egr1* in TKO MEFs reconstituted with wild-type Elk-1 (red), Elk-1^FW^ (blue), Elk-1^nonA^ (purple), or with the empty pMY vector control (black). (C) Signal-induced changes in H3S10ph and H3K9acS10ph at the 5′ flanking and transcribed sequences of *Egr1, Egr2*, *Fos*, and *Ier2* in TKO MEFs reconstituted as in (B). Data are means ± SEM, n = 3. ^∗^p < 0.05 by t test compared with TKO MEFs with empty vector. See also Figures S5D and S5E.

We used the TCF Elk-1 mutants to test the role of the TCF activation domain in establishment of histone modifications at direct TCF-SRF target promoters. Consistent with the genomic data, TPA stimulation induced TCF-dependent changes in H3S10ph, H3K9acS10ph, H4K16ac, H3K27ac, H3K9acK14ac, and H3K4me3 at *Egr1*, with the changes in H3K9acK14ac and H3K4me3 being more pronounced in the transcribed sequences (Figure 5A; see Figure S1). Re-expression of wild-type Elk-1 restored induction of the histone modifications and recruitment of the transcriptional machinery including Mediator, Cdk9, and PolII (Figures 5B and S5C). In contrast, the transcriptionally inactive Elk-1^nonA^ mutant restored neither TPA-induced Mediator, Cdk9, and PolII recruitment nor the increases in histone modifications, indicating that the latter are associated with transcription (Figures 5B and S5D). However, the transcriptionally inactive Elk-1^ΔFW^ mutant significantly restored TPA-induced increases in H3S10ph and H3K9acS10ph in the vicinity of the upstream *Egr1* TCF-SRF sites (Figures 5B and 5C). Similar results were observed at three other direct TCF target genes, *Egr2*, *Fos*, and *Ier2* (Figures 5C and S5E). Taken together, these results indicate that induced H3 phosphorylation requires only receipt of signal by TCF, while induction the other modifications requires that TCF be able to recruit the transcriptional machinery (see Discussion).

### Ordered TCF-Dependent Histone Modifications at *Egr1*

**Figure 6:**
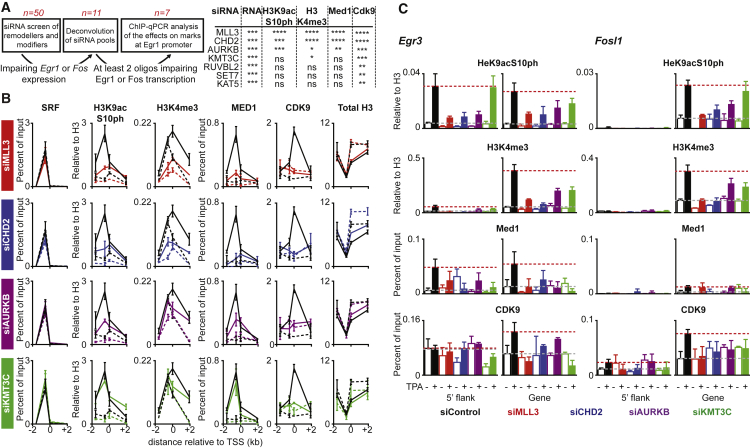
siRNA Screen Defines an Ordered Series of TCF-Dependent Chromatin Steps Required for *Egr1* and *Fos* Activation (A) Left: siRNA screening strategy, with number of candidate hits remaining at each stage indicated. Right: summary of siRNA effects on transcription or ChIP signals at the 5′-flanking or TSS-proximal region of *Egr1* (one-way ANOVA, corrected for multiple comparisons: ^∗∗∗^p < 0.001, ^∗∗^p < 0.01, ^∗^p < 0.05; ns, not significant). (B) Quantitative ChIP at *Egr1* with the indicated antibodies following depletion of MLL3 (red), CHD2 (blue), AURKB (purple), and KMT3C (green) or scrambled oligonucleotide control (black). Histone ChIP signals were normalized to H3. (C) Quantitative ChIP at *Egr1*, *Egr3*, and *Fosl1* analyzed using 5′-flanking or TSS-proximal PCR probes (see Figure 4A) following depletion of MLL3 (red), CHD2 (blue), AURKB (purple), and KMT3C (green) or scrambled oligonucleotide control (black). Histone ChIP signals were normalized to H3. Data are mean ± SEM; n = 3.

To gather further insight into the interdependence of the histone modifications accompanying TCF-dependent transcriptional activation, we screened small interfering RNAs (siRNAs) targeting 50 catalytic subunits of chromatin-modifying and remodeling complexes (Fazzio et al., 2008) for their ability to inhibit accumulation of *Egr1* and *Fos* pre-mRNA or mRNA following TPA stimulation (Figure 6A; Table S4). The screen identified the methyltransferases KMT3C, MLL3, and SET7; two helicases, the chromodomain protein CHD2 and the RUVBL2 subunit of the Ino80 and TIP60 complexes; the Aurora B histone kinase, AURKB; and the MYST-family acetyl-transferase KAT5 (Figures 6A and S6).

We used ChIP to assess the roles of these factors in TPA-induced activation of the TCF direct target genes *Egr1*, *Egr3*, and *Fosl1*. MLL3 and SET7 were detectable by ChIP at *Egr1*, the latter in a TPA-inducible manner, and are therefore likely to act at the promoter (Figure S7A). Depletion of SET7, KAT5, KMT3C, and RUVBL2 did not affect induced Mediator recruitment, indicating that signaling to the TCFs is intact in depleted cells, but blocked Cdk9 recruitment (Figure S7B). Finally, AURKB depletion inhibited TPA induction of *Egr1* and *Fos* but did not affect induction of *Acta2* or *Ctgf* by serum or cytochalasin D (Figure S7C). None of the siRNAs affected serum induction of *Srf*, *Acta2*, and *Cyr61* in NIH 3T3 cells (data not shown).

At *Egr1*, depletion of MLL3 or CHD2 reduced the basal level of H3K4me3 and the TPA-induced H3 phosphoacetylation and K4 trimethylation (Figures 6B and S7D). In contrast, depletion of AURKB reduced both modifications, while KMT3C depletion impaired only induction of H3K4me3 (Figure 6B). Depletion of MLL3, CHD2, and AURKB also inhibited Mediator recruitment (Figure 6B). Similar results were obtained at *Egr2* and *Fosl1*, although Mediator recruitment was not detectable at *Fosl1* with the probes used. Taken together, these results suggest that MLL3, CHD2, and AURKB act prior to KMT3C in histone modification and transcription initiation at TCF target genes, at a step prior to PolII engagement.

**Figure 7:**
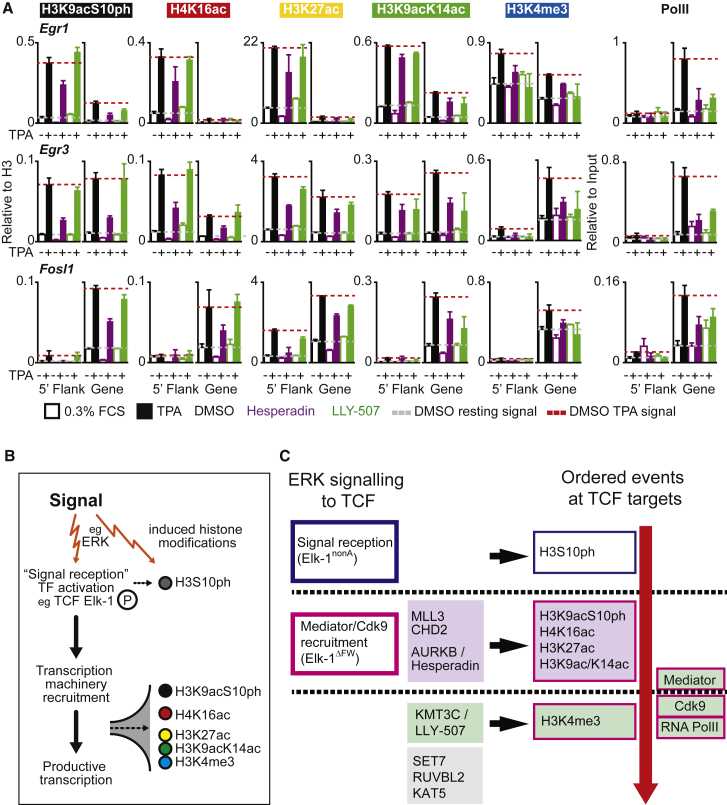
Acute Inhibition of AURKB and KMT3C Inhibits IE Gene Transcription (A) Quantitative ChIP with the indicated antibodies at *Egr1*, *Egr3*, and *Fosl1* analyzed using 5′-flanking or TSS-proximal PCR probes (see Figure 4A) upon TPA stimulation with 250 μM hesperadin (purple), 1 μM LLY-507 (green), or DMSO vehicle (black). Histone signals were normalized to H3. Dashed red line, TPA-induced level; solid yellow line, resting level in vehicle-treated cells. Data are mean ± SEM; n = 4. (B) Signal-induced transcription factor activation and histone modification. Dotted arrows indicate that inducible histone modifications depend on signal reception and recruitment of the transcription machinery by TCF Elk-1. (C) Signaling and activation of direct TCF-SRF target genes. Left: role of TCFs and chromatin modifiers. MLL3 and CHD2 are placed above AURKB, as they affect both basal and induced levels of H3K4me3. Right: sequence of events at the promoter. Box outlines indicate dependence on TCF functions; shading indicates dependence on chromatin modifiers. We did not assess the dependence of H4K16ac, H3K27ac, or H3K9acK14ac on CHD2 and MLL3. AURKB is an H3S10 kinase but may act indirectly, via a factor required both for S10ph and the other modifications. Similar results were obtained at *Egr1*, *Egr3*, and *Fosl1*, although Mediator recruitment was not detected on *Fosl1* and was dependent on KMT3C at *Egr3*.

We also tested whether acute inhibition of Aurora B and KMT3C affected TPA-induced histone modifications at *Egr1*, *Egr3*, and *FosL1*. Hesperadin is an indolinone inhibitor of Aurora-B (half-maximal inhibitory concentration [IC_50_] = 250 nM; de Groot et al., 2015), while LLY-507 is a potent inhibitor of KMT3C (IC_50_ < 15 nM, >100-fold selectivity over other MTs; Nguyen et al., 2015). Short pretreatments with either compound inhibited TPA-induced PolII recruitment and histone modifications at *Egr1*, *Egr3*, and *Fosl1* (Figure 7A). These results support the notion that the siRNA depletion results reflect direct effects on cell signaling rather than indirect effects of long-term protein depletion. Taken together with the TCF mutant data, these results provide genetic evidence that at direct TCF-SRF target genes, histone modifications proceed in a specific order, dependent on signal reception by the TCFs and consequent recruitment of the transcriptional apparatus (Figure 7B; see Discussion).

## Discussion

We used an unbiased approach to study the relationship between TPA-induced ERK signaling, changes in histone modification, and activity of the ERK-regulated TCF transcription factors. Within 30 min of TPA stimulation, more than 2,000 TSS regions exhibited increased histone modifications, which exhibited a continuous and nested hierarchical relationship, which thus appears to be a general feature of gene activation. We found that most of the ERK-induced chromatin response in MEFs is dependent on the TCF family of SRF cofactors and requires that they be competent both to receive signal and to activate transcription.

### A Common Pattern of TPA-Induced Histone Modifications

TPA-induced changes were ordered as H3K9acS10ph, H4K16ac, H3K27ac, H3K9acK14ac, and H3K4me3. The increases in H3K4me3 and H3K9ac14ac occur preferentially in transcribed sequences, suggesting that they reflect transcription itself. The magnitude of the TPA-induced change in histone modifications in TSS regions correlates remarkably well with the increase in transcription of their associated genes (see also Gualdrini et al., 2016). Thus, although H3K4me3 and H3K9ac14ac are known to be associated with transcriptional activity (Benayoun et al., 2014, Chen et al., 2015, Guenther et al., 2007, Ng et al., 2003), quantitation of the difference between two cell states is likely to provide a good measure of transcriptional differences between them.

We also detected TPA-induced changes in histone modification at more than 1,000 putative remote regulatory sites, defined by published DNase I HS data sets. These changes exhibited a similar hierarchical relationship to changes at TSS regions, although the increases in H3K9acK14ac and H3K4me3 were much lower. It is likely that activation of these sites occurs in association with activation of linked distant genes.

The greatest TPA-induced change in histone modification was H3 phosphoacetylation. Phosphorylation of H3 at serines 10 and 28 was classically correlated with inducible gene activation (Anest et al., 2003, Clayton et al., 2000, Mahadevan et al., 1991, Soloaga et al., 2003, Yamamoto et al., 2003, Zhong et al., 2001). Although the antibody we used does not discriminate between H3K9acS10ph and H3K27acS28ph, both of these modifications occur at immediate-early genes in MEFs (Soloaga et al., 2003, Zhong et al., 2001). Our data suggest these modifications may generally occur at inducible genes. H3 phosphorylation is thought to promote chromatin de-compaction and to facilitate chromatin regulatory events (for review, see Healy et al., 2012). For example, targeting of the H3 kinase MSK directly to promoters can induce both H3 phosphorylation and gene transcription (Lau and Cheung, 2011), and recent studies have shown that H3S28 phosphorylation potentiates transcription on chromatin templates in vitro (Josefowicz et al., 2016).

Although these results suggest H3 phosphorylation is a feature of all gene induction, we cannot rule out the possibility that it occurs only where ERK is also activated. For example, steroid hormones direct activate the ERK pathway (for references, see Vicent et al., 2006), and in many cell types, a basal level of ERK signaling results from stochastic triggering of the pathway (Aoki et al., 2013).

### Histone H3 Phosphorylation Is an Early Step in Gene Activation

A simple explanation for the hierarchy observed in the genomic analysis is that it reflects the kinetics of each modification. Genomic analysis indicated that H3 phosphoacetylation indeed preceded the other TPA-induced histone modifications at TSS, with H3K4me3 accumulation in transcribed sequences occurring at later times. Although technical considerations precluded genomic analysis of H3S10ph induction at TSS, it clearly preceded H3 phosphoacetylation at remote regulatory sites. Analysis of specific genes confirmed the genomic data: H3S10 phosphorylation clearly preceded all the other TPA-induced histone modifications both at direct TCF-SRF target genes, consistent with a previous study of *Egr1* (Riffo-Campos et al., 2015), and also at TCF-independent TPA-inducible genes. Taken together, our results support the notion that the hierarchy of changes in histone modifications observed in genomic analysis reflects the temporal order of their induction.

Our data do not provide further insight into the potentially distinct locations and functions of H3S10ph and H3S28ph nucleosome populations (Josefowicz et al., 2016; reviewed by Healy et al., 2012); however, the continuous distribution of the H3K9acS10ph ChIP-seq signal suggests that both S10 and S28 phosphoacetyl H3 populations are similar, at least at the time point analyzed. Although we explored only time points leading up to the maximal transcriptional activation of direct TCF-SRF target genes, 30–45 min after stimulation, it will be interesting to see how modifications change as transcription returns to baseline levels.

### The TCFs Are Required for Most TPA-Induced Histone Modification

A central question in inducible chromatin modifications has been the role played by specific transcription factors in targeting of modifications and their relation to transcription itself. Our analysis of MEFs lacking all three TCFs demonstrated that most TPA-induced histone modification changes were TCF dependent, as was the transcriptional response itself (Gualdrini et al., 2016). We found that the hierarchical relationship of TPA-induced changes in histone modifications at direct genomic TCF-SRF targets, identified by an integrated ChIP-seq/Hi-C approach, was substantially identical to that seen at TCF-independent TSSs. These observations again support the idea that the hierarchy of changes in induced histone modification reflects a general process underlying gene activation per se, rather than events specific to particular transcription factors. Indeed, many different transcription factor binding motifs are enriched at DNase I HS regions that exhibit inducible histone modifications.

Experiments with TCF Elk-1 mutants showed that induction of all the histone modifications examined is dependent on the ability of TCF to receive its activating signal (i.e., its phosphorylation) (Figure 7B). Thus, although Elk-1 can recruit its activating kinase ERK to DNA (Göke et al., 2013, Zhang et al., 2008), inducible histone modifications must require additional events associated with TCF activation. Experiments with the Elk-1^ΔFW^ mutant, which can be phosphorylated but cannot recruit the transcriptional machinery (Balamotis et al., 2009), show that histone modifications downstream of H3S10ph proceed effectively only when TCF is competent to activate transcription (Figure 7B). This suggests a model in which unacetylated H3S10ph (and possibly H3S28ph) at TSSs is an indicator of signal reception, with the other modifications arising upon activation of transcription. Although we cannot exclude the possibility that H3S10ph and the other histone modification events occur independently, relying on a common upstream regulator, we consider this unlikely.

The TCFs also provide the primary input for signals at putative “enhancer” SRF sites associated with *Egr1*, *Egr3*, and *Fosl1*, even though eRNA synthesis may facilitate activation (Kim et al., 2010, Schaukowitch et al., 2014). Because induction of chromatin modifications at TSS that are not direct TCF-SRF genomic targets is also TCF dependent, we speculate that TCF-dependent histone modification at these genes will reflect signaling to transcription factors whose expression requires TCF (Gualdrini et al., 2016).

### TPA Induction of Direct TCF-SRF Targets Requires Multiple Cofactors

We used siRNA screening to identify candidate regulatory cofactors for histone modifications at direct TCF-SRF genomic targets (Figure 7B; see also Gualdrini et al., 2016). Depletion of chromatin regulators required for H3K9acS10ph phosphorylation prevented induction of the other modifications (Figure 7C). Our data suggest direct involvement of AURKB in IE gene activation, but we were unable to detect it by ChIP, and it remains possible that its acts indirectly. The identity of the kinase(s) mediating H3S10ph at IE genes has been controversial (for review, see Healy et al., 2012). A recent study implicated AURKB in interphase H3S28 phosphorylation in B cells (Frangini et al., 2013). MSK1/2 and PIM1 have also been implicated in H3S10 phosphorylation at *Egr1* and *Fosl1* (Soloaga et al., 2003, Zippo et al., 2007), but the MSKs did not score in our screen, and we note that MSK1/2-null MEFs exhibit no defect in TPA-induced *Egr1* or *Fos* induction (Wiggin et al., 2002). It is very likely that there is some gene or stimulus specificity in the kinases mediating H3S10ph. For example, in our experiments AURKB depletion did not block induction of *Acta2* and *Ctgf* by serum or cytochalasin D, while others have shown that in MEFs, IKKα is responsible for TNFα-induced H3S10ph at the IκB promoter (Anest et al., 2003, Yamamoto et al., 2003).

MLL3 and CHD2 promote basal levels of H3K4me3, and together with AURKB are required at an early stage for TCF-dependent signal-induced induction of all modifications (Figure 7C). The MLL3/4 methyltransferase complexes control H3K4 methylation at enhancers (Hu et al., 2013), but MLL3 is detectable by ChIP at *Egr1*, suggesting that it may act directly to influence H3K4me3 accumulation there. CHD2 is found at many TSS regions (Ernst and Kellis, 2013, Siggens et al., 2015) and may be required for signal-induced nucleosome remodeling events, which are known to accompany IE gene activation (Riffo-Campos et al., 2015).

Four regulators, SET7, RUVBL2, KAT5, and KMT3C, apparently act at a late stage in activation of TCF direct target genes, being required for signal-induced recruitment of Cdk9, but not Mediator recruitment (Figure 7B). The SET7 H3K4 methyltransferase, implicated in signal-regulated transcription activation (Keating and El-Osta, 2013), exhibits signal-induced recruitment to *Egr1* and is therefore likely to act directly. RUVBL2 and KAT5 are both found in the TIP60 acetyltransferase complex (Lu et al., 2009), but recent studies suggest that KMT3C acts predominantly on non-histone substrates (Olsen et al., 2016); further work will be required to elucidate their roles.

### Conclusions

We identified a hierarchy of ERK-induced histone modifications at TSSs in MEFs that accompanies activation of transcription. At direct targets of the TCF-SRF transcription factor complex, early H3 phosphorylation and phosphoacetylation is dependent on TCF phosphorylation and precedes the establishment of further modifications, which in turn depend on the ability of the TCFs to activate transcription. This modification cascade appears to be a general feature of gene activation, because it also occurs at TCF-independent TSSs. It remains to be seen to what extent the regulatory enzymes involved are pathway or gene specific, and our future work will focus on this question.

## STAR★Methods

### Key Resources Table

**Table:** 

REAGENT or RESOURCE	SOURCE	IDENTIFIER
**Antibodies**		

H3K9acS10ph	Abcam	Ab12181-50, Lot# 770587
H4K16ac	Abcam	Ab61240, Lot# GR284-15; RRID: AB_941967
H3K27ac	Abcam	Ab4729, Lot# GR144577-2; RRID: AB_2118291
H3K9acK14ac	Millipore	06-599, Lot# 1969119; RRID: AB_2115283
H3K4me3	Abcam	Ab1012, Lot# GR56209-1; RRID: AB_442796
H3S10ph	Millipore	04-817; RRID: AB_1163440
H3	Abcam	AB1791; RRID: AB_302613
Med1	Bethyl Lab	A300793A
Pol II 8WG16	Santa Cruz	sc-56767; RRID: AB_785522
CDK9	Santa Cruz	sc-484; RRID: AB_2275986
SRF	Santa Cruz	sc-335; RRID: AB_2255249
Elk-1	In-house	N/A

**Chemicals, Peptides, and Recombinant Proteins**		

12-O-Tetradecanoylphorbol-13-acetate (TPA)	Sigma	P1585
Hesperadin	Stratech Scientific	S1529
Lipofectamine RNAiMax	Invitrogen	13778030
Dynabeads Protein G	Invitrogen	10009D
LLY-507	Cambridge Bioscience	16441

**Critical Commercial Assays**		

SuperScript III Reverse Transcriptase (RT) Kit	Invitrogen	11754250
TruSeq ChIP Sample Prep Kit	Illumina	IP-202-1012

**Deposited Data**		

Raw and processed data - ChIP-seq analysis of histone modifications, TPA-stimulated wildtype and TKO MEFs	This paper	GEO: GSE75002
DNA’se I HS sites from NIH and MEFs	https://www.ncbi.nlm.nih.gov/geo/	GEO: GSM1003831 and GSM1014199
Raw and processed data - RNA-seq data, TPA-stimulated wildtype and TKO MEFs	Gualdrini et al., 2016	GEO: GSE75667
Raw and processed data - SRF and Elk-1 ChIP-seq data, TPA-stimulated wildtype and TKO MEFs	Gualdrini et al., 2016	GEO: GSE75667

**Experimental Models: Cell Lines**		

Mouse Embryonic Fibroblasts (MEF) wild type	Gualdrini et al., 2016	N/A
TKO MEF	Costello et al., 2010, Gualdrini et al., 2016	N/A
TKO MEF + pMY-Empty	Gualdrini et al., 2016	N/A
TKO MEF + pMY-Elk-1 wt	Gualdrini et al., 2016	N/A
TKO MEF + pMY-Elk-1 nonA	Gualdrini et al., 2016	N/A
TKO MEF + pMY-Elk-1 FW	Gualdrini et al., 2016	N/A

**Sequence-Based Reagents**		

RT-PCR Primers	This paper	see Table S3
SMARTpool ON-TARGETplus siRNA	Dharmacon	see Table S4
Individual ON-TARGETplus siRNA	Dharmacon	see Table S4

**Software and Algorithms**		

R programming language	R Development Core Team, 2015. R: A language and environment for statistical computing. R Foundation for Statistical Computing, Vienna, Austria.	https://www.R-project.org/
Deseq	Anders and Huber, 2010	http://bioconductor.org/packages/release/bioc/html/DESeq.html
Samtools	Li et al. (2009)	http://samtools.sourceforge.net/
BWA	Li and Durbin (2010)	http://bio-bwa.sourceforge.net/
Bedtools	Quinlan laboratory at the University of Utah	http://bedtools.readthedocs.io/en/latest/
Prism 6	GraphPad Software	http://www.graphpad.com/scientific-software/prism/
Homer	HOMER (Heinz et al., 2010)	http://homer.salk.edu/homer/motif/

**Other**		

Scaling factors calculation for ChIP-seq samples comparison	This paper	See STAR Methods
Regression models between RNA and histone mark fold changes	This paper	See STAR Methods
Distance Index for chromatin modification hierarchical clustering	This paper	See STAR Methods
Estimation of dependency of changes on cell line background	Gualdrini et al. (2016)	See STAR Methods

### Contact for Reagent and Resource Sharing

Further information and requests for reagents may be directed to and will be fulfilled by the Lead Contact, Dr. Richard Treisman (richard.treisman@crick.ac.uk).

### Experimental Model and Subject Details

Mouse Embryonic fibroblasts (MEFs) were derived from either wild-type mice (WT) or mice lacking the Elk-1, Net and SAP-1 TCFs (Elk1^−/−^ Elk3^δ/δ^ Elk4^−/−^; TKO MEFs) and immortalized by infection with retrovirus expressing SV40 large T protein (Costello et al., 2010). MEFs were cultured in DMEM (GIBCO, Invitrogen) supplemented with 10% FCS at 37°C and 10% CO_2_. Cells were maintained in 0.3% FCS for 48 hr, then stimulated with 12-O-Tetradecanoylphorbol-13-acetate (50ng/ml) for 30 min or as indicated in the Figure legends. TKO cells were reconstituted with wild-type human Elk-1, or the transcriptionally inactive Elk-1^nonA^ and Elk-1^ΔFW^ mutants using retrovirus infection (see Gualdrini et al., 2016). For some experiments cells were pretreated for 30 min with 250nM Hesperadin or 1 μM LLY-507.

### Method Details

#### siRNA screen

ON-TARGETplus SMARTpool siRNAs (Dharmacon) were used to screen 50 catalytic subunits of chromatin-modifying and remodelling complexes (Fazzio et al., 2008; see Table S3) for inhibition of TPA-induced *Egr1* and *Fos* expression. MEFs were transfected with 20 nMol each siRNA pool using RNAimax (Invitrogen) for 72 hr. *Egr1* and *Fos* transcription was monitored at the pre-RNA or mRNA level before and after TPA stimulation in biological triplicates by qRT-PCR (primers in Table S3). An inhibition threshold was set at 80% of the level seen in untreated cells, at p ≤ 0.05, based on the inhibition observed using SRF siRNA as a positive control. siRNA pools inhibiting at least 3 out of the 4 transcription readouts and from which at least two siRNA oligonucleotides inhibited induction (at 20nMol each), were analyzed further. Screening results are summarized in Table S4.

#### Chromatin immunoprecipitation

ChIP was performed as described (Esnault et al., 2014, Miralles et al., 2003), with the following modifications: fixation was stopped by the addition of 250 mM glycine, sonication was performed with a Bioruptor Plus and magnetic G protein beads (Invitrogen) were used for recovery. SYBR Green-based real-time PCR (Invitrogen) was performed using dilutions of genomic DNA solution for calibration and to derive arbitrary abundance units. For primers see Table S3. For combination with siRNA treatments, cells were transfected with siRNAs as above for 72 hr prior to processing for ChIP.

#### Antibodies

Antibodies used were: H3K4me3 (AB1012, Abcam); H3K9ACS10P (AB12181X510/AC, Abcam); H4K16Ac (AB61240, Abcam); H3K9K14AC (06-599, Millipore); H3S10P (04-817, Millipore), H3S10P (C15410116/pAb-116-050, Diagenode); H3K27Ac (AB4729, Abcam); Histone H3 (AB1791, Abcam); MED1 (A300793A, Bethnyl Lab); SRF (sc-335, S. Cruz); CDK9 (sc-484, S. Cruz); Pol II 8WG16 (sc-56767, S. Cruz). Elk-1 anti-mouse Elk-1 aa309–429 was made in-house and re-purified against a peptide derived from the human Elk-1^nonA^ protein (aa309-429) (Costello et al., 2010). The H3K9acS10ph antibody cross-reacts with the mitogen-inducible H3K27acS28ph epitope and with H3K9acS10ph acetylated at K14; it does not cross-react with non-phosphorylated acetyl-H3 (Rothbart et al., 2015). The H3S10 antibody does not cross-react with H3K9acS10ph, or H3S28ph (Rothbart et al., 2015). The H3K27Ac antibody also cross-reacts with acetylated H3 K9, K14, K18 and K27 (Rothbart et al., 2015), but the signal obtained is substantially symmetric across TSS regions, distinct from the K9ac14ac signal. This antibody has been used extensively to assess many different cell types, and is included for comparison.

#### Chip-Seq sample preparation

DNA samples were end repaired, poly-A tailed and Illumina single-end adapters ligated following the standard Illumina protocol with minor adjustments. Agencourt AMPure XP beads at 0.8x ratio were used to remove adaptor dimers after ligation. The Illumina kit Phusion enzyme was replaced by Kapa HiFi HotStart ready mix. Post PCR, AMPure XP beads were used at a 1:1 ratio to maintain size integrity. DNA fragments size-selected and purified from a 2% agarose gel using the QIAquick gel extraction kit, quality controlled on the DNA 1000 BioAnalyser 2100 chip. Sequencing was on the Hi-seq2500 to generate 150 base read lengths.

#### ChIP-Seq data processing

All ChIP-Seq datasets were cropped to 50 bp and aligned using BWA to the mouse genome (mm9) with the default settings. All ChIP-seq mapped read counts were normalized to 30 million total aligned reads. All annotated Refseq genes were assessed for histone mark coverage across a 3kb window spanning their TSS (−2kb to +1kb; the “TSS region”). This window was chosen to minimize overlap with other regulatory features without compromising identification of changes using DESeq. For DNase I HS sites chromatin changes occurring with ± 2kb windows of the peak summit were analyzed.

#### Scaling Factors

To control for technical variation across conditions, we calculated scaling factors relative to one condition for each antibody (MEF_03 replica 1). Scaling factors were retrieved using two independent methods which gave virtually identical results, as assessed by linear regression between normalized samples, which exhibited a slope of 1.00 ± 0.01 and spearman R^2^ ∼1.0. The complete list of samples and replicates and the normalization factor used are given in Table S1. Data presented in the paper were normalized using Scaling Method 1.

##### Method 1

Sequencing read depths across the TSS region were normalized to those of a set of genes which are actively transcribed, and invariant across all the experimental conditions used in the study, which was defined as follows. Invariant expressed genes were defined as those fulfilling the following criteria: (i) they showed at least 6 intronic RNA-seq reads; (ii) readcounts were statistically not significantly different across all conditions, as assessed by DESeq; (iii) the standard deviation across all conditions was less than 15% of the mean. 487 genes fulfilled these criteria.

##### Method 2

This method assumed a quasi-normal distribution of invariant TSS region readcounts. The ChIP-seq signal of the histone modifications in each sample was expressed relative to the signal e in MEF_03_replica_1. Since the distribution of invariant TSS region readcounts should be approximatively Gaussian, we obtained the μ_diff_ and σ_diff_ parameters of the best-fit Gaussian using the dnorm() function in R and optimized by maximum likelihood with the optim() function. Invariant TSS regions were defined as those with differences in read counts within 1σ from the mean difference (μ_diff_). This method was used to carefully scale samples of the time course (0, 5 and 15 following TPA stimulation). To directly compare also the 30 min time point (which was part of an independent library preparation and sequencing flow) with these earlier time points, we re-scaled the sample against the signal in the resting condition for the H3S10ph, H3K9acS10ph and H3K4me3 antibodies. For H3S10ph, the maximal signal was detected at DNase I HS, the signals at TSS being weak, so we therefore used the DNase I HS signal to define the scaling index. Scaling indexes are provided both in GEO: GSE75002 and in Table S1.

#### Analysis of ChIP-seq histone modification data

For each antibody, normalized readcounts across all RefSeq (release 63) TSS regions in cells maintained in 0.3% FCS or stimulated with TPA for 30’ (each condition run in duplicate) were compared using DESeq (Anders and Huber, 2010). For TSS analysis, the minimum read threshold was set at the 25th percentile of the readcount distribution for each antibody, allowing unequivocal operation of DESeq. Changes of > 20% with associated p < 0.05 were deemed significant. ∼12000 TSS regions generated ChIP-seq signals above this detection threshold. For DNase I HS analysis, the TSS threshold proved too low for reliable use of Deseq, since many of the ∼290000 DHS sites showed insufficient read coverage. To identify an appropriate threshold, we pooled data from all 5 antibodies, assessed the DNase I HS readcount distribution, and chose a minimum read threshold such that the Deseq dispersion plot was indicative of an homogeneous population. A threshold of 250 read counts per 4kb window (5x the 25th percentile) identified ∼50000 DHS which allowed reliable operation of DeSeq. Changes of at least 2-fold and associated adjusted p < 0.01 were considered significant.

#### Metaprofiles

For each histone modification, the normalized average readcount density per nucleotide, using a 51bp smoothing window, was used to generate a density plot centered on the RefSeq TSS. Read densities over 150bp on each side of the TSS were removed to reduce distortion of the profiles by the low readcounts at nucleosome-depleted TSS regions. Difference profiles display the difference between the readcount density per nucleotide of the resting and TPA-stimulated samples across the TSS regions.

#### Histone mark hierarchical Clustering

We used hierarchical clustering to organize TPA-induced changes in each histone modification at TSS regions. For each modification the fold-change upon TPA stimulation at each TSS region was calculated. A Euclidean distance matrix across the 5 modifications was applied to the R agglomerative hierarchical clustering method hclust (using the single linkage, “friends of friends’ clustering strategy, closely related to the minimal spanning tree). The nesting pattern generated by hclust was validated through multi-scale bootstrap re-sampling by pvclust (Suzuki and Shimodaira, 2006) in the R package. In pvclust the AU p-value, calculated using multiscale bootstrap resampling, is superior to the BP p-value calculated by ordinary bootstrap resampling. The method was applied to define relationships between (i) all 2364 TSS regions exhibiting a change in response to TPA in wildtype MEFs (see Figure S2); (ii) TSS regions divided into subsets in which any 1,2,3, or 4, or all 5 modifications change upon stimulation as assessed by DESeq (see Figure 1A); each subset gives the same pattern; (iii) TCF-independent, TCF-dependent and Elk-1 direct target TSS regions (see Figure 3).

We also attempted to cluster the TSS regions themselves according to the pattern of relative changes in the 5 modifications. To minimize asymmetry, the TSS regions were separated into subsets as above according to the number of histone modifications that significantly changed upon stimulation. A Euclidean distance matrix was then generated per group of TSS regions. The distance matrix was applied to the R agglomerative hierarchical clustering method hclust, using the ward.D2 linkage method which minimizes the total within-cluster variance (Murtagh and Legendre, 2014). Hierarchical clustering of TSS regions did not present significant obvious groups while the main effect observed was the relation between histone mark inductions.

#### Distance Index

To assess the potential interdependence of the different histone modifications, we calculated a distance index (DI), defined as the average difference between the degree of TPA-induced increase in a given modification (the “leading mark”; x), and that for any other modification (y). For any histone modification that exhibits a positive change x_i_ > 1, we calculated the cumulative difference DI from any other modification, y_i_. Thus, for the TSS regions with x > 1, DI = [Σ(x_i_-y_i_)]/n for i = 1 to n. The DI values for any paired comparison were annotated into a matrix (see Figures 1C and S2). This method orders the modifications in the same way as the hierarchical clustering, and reveals two subclusters: H3K9acS10ph and H4K16ac, and H3K27ac, H3K9acK14ac and H3K4me3.

#### TCF-dependence of TPA-induced ChIP-seq signal changes

ChIP-seq signals in wild-type and TKO MEFs, and TKO MEFs reconstituted with different Elk-1 mutants, were compared using the iterative method described previously (Gualdrini et al., 2016), which compares the degree of induction of each histone modification under two different conditions and identifies genes that are similarly affected by background. The basic assumption of the method is that activity of genes dependent on a particular transcriptional regulator will be similarly affected by changes in abundance or activity of that regulator. It does not require paired conditions, but does not allow statistical conclusions to be drawn regarding particular genes, although it can give an overall estimate of the numbers of genes subject to shared control. Data are presented as TPA-induced fold change in one background (x axis: fold change in background 1), versus the difference between the fold change in that background and a second background (y axis: [fold-change in background 1]-[fold-change in background 2]). Systematic influence of the second background will generate a distribution asymmetrically disposed about y = 0, and linear regression analysis of data points above and below y = 0 should yield slopes that are significantly non-zero.

#### Motif analysis

Transcription factor motif analysis was performed using HOMER (Heinz et al., 2010). Sequences ± 100 bp from the peak summit of DNaseI HS were analyzed; all DNaseI HS were used as background.

#### Regression models between RNA and histone mark fold changes

To investigate whether TPA-induced changes in histone modification could be used to model the resulting changes in gene transcription, we performed both linear and multiple regression analysis.

For linear regression, we calculated (i) the readcount ratio between TPA-stimulated and un-stimulated conditions (TPA / 0.3%FCS) in wild-type MEFs for each histone modification, at all TSS regions where at least one modification increased following stimulation (n = 2364); and (ii) the fold increase in transcription, defined as the ratio of intronic reads in TPA-stimulated and un-stimulated conditions. For genes lacking intronic reads, the ratio of all reads aligned to their ORF was used. To assess whether the dataset needed to be scaled, we assessed the skewness for each histone modification and the RNA fold-change using the skewness function from the e1071 package in R. The dataset was significantly skewed, and a Log2 transformation was therefore applied to each variable allowing us to investigate the log-log regression between each individual mark (or incombinations) and the RNA fold changes. The coefficients of a log-log regression are easily interpretable: since log(y) = β_0_ + β_1_^∗^log(x) + ε, a change of 1% in x will lead to a β_1_% change in y. Linear regression between RNA changes and each mark, considered separately, was done using R (see Figure S5).

Multiple-linear regression was performed using stepwise model selection by AIC (Akaike information criterion) which measures the relative quality of statistical models developed for a given set of data. Models in which histone modifications were considered to be independent of or dependent on each other were developed using the stepAIC function of the MASS package in R (Venables and Ripley, 2002) (see Figure S3). This approach mitigates collinearity between variables. For each model quality was assessed by evaluating diagnostic plots generated in R: “Residuals vs Fitted” detects non-linearity, unequal error variances, outliers and non-homogeneous dispersion; “Q-Q plot” compares probability distributions between the two sets of data; “Residual vs Leverage” assesses the influence of each observation on the regression coefficients (Cook’s distance statistic is a measure of the extent of change in the model estimates when a particular observation is omitted) (see Figure S3).

### Quantification and Statistical Analysis

See Method Details for details of statistical analysis.

### Data and Software Availability

#### Software

See Key Resources Table.

#### Data Resources

The raw sequencing files and the processed data are available at the following databases:

The accession number for the ChIP-seq analysis of Ras/ERK signal-induced histone modifications in mouse embryo fibroblasts reported in this paper is GEO: GSE75002.

The accession number for the RNA-seq analysis of Ras/ERK signal-induced transcriptional changes and the ChIP-seq analysis of SRF and TCF DNA-binding profiles in mouse embryo fibroblasts reported in this paper is GEO: GSE75667.

## Author Contributions

C.E. and F.G. designed, conducted, and interpreted experiments and wrote the paper. F.G., S.H., G.K., P.E., and A.S. analyzed genomic data and wrote scripts. N.M. performed sequence analysis. R.T. conceived the project, suggested and interpreted experiments, and wrote the paper with F.G. and C.E.

## Supplemental Information

Document S1. Figures S1–S7

Table S1. ChIP-Seq Runs, Related to Figures 1, 2, and 3 Summary of the ChIP-seq runs, listing Run name, Cell source, Genotype and/or transfectant, Antibody data, and scaling factors. Data are available at GEO: GSE75002.

Table S2. Summary of Histone Modification ChIP-Seq Data at TSS Regions and DNAse I HS, B, Related to Figures 1, 2, 3, 4, S3, and S4 (A) TPA-induced changes occurring in TSS regions Columns 1–6. Gene description: Official gene symbol; transcript ID; Genomic coordinates (Chromosome, Strand, Start and Stop). Columns 7–10. Relationship between each TSS and the 2577 SRF ChIP-seq peaks, including closest SRF peak coordinate; distance to the closest peak; whether the TSS is linked to SRF by Hi-C; Direct targets for TCF-SRF histone regulation defined those TSS exhibiting TPA-induced histone modification located 10 kb of an SRF peak and/or linked to one by Hi-C (n = 817). Columns 11–16. Summary of the RNA-seq data (Gualdrini et al., 2016) including TPA-induction (0 or 1); Direct TCF-SRF targets, as assessed by comparison of RNA-seq and integrated SRF ChIP-seq / Hi-C data (n = 763); total and intronic RNA read counts in wild-type MEF before and after 30' TPA stimulation. Columns 17–22. Effect of 30 minute TPA stimulation on each histone modification. TPA-induction (0 or 1). Columns 23–28. Dependence of each induced histone modification on TCF (0 or 1) obtained by comparison of wildtype and TKO MEFs using regression method. Columns 29–34. Restoration of induced histone modifications (0 or 1) by expression of wildtype Elk-1 in TKO MEFs, assessed by regression method. Columns 35–40. Restoration of induced histone modifications (0 or 1) by expression of Elk-1 activation-domain mutants in TKO MEFs, assessed by regression method. Columns 41–100. Normalized and averaged histone modification read counts in resting or TPA-stimulated (30') cells, for each of the 5 antibodies. Columns 101–108. Normalized and averaged read counts for each of the 5 histone modifications in resting or TPA-stimulated (30') cells. (B) Changes occurring at the DNAse I HS sites. Column 1–4. Coordinates for all DNAse I HS sites which show TPA-induced change in at least one histone modification (n = 2404). Columns 5–8. Relation of each DNAse I HS site to SRF ChiP-seq peaks and closest TSS. Number of SRF peaks coincident with the DNAse I HS region (peak ± 2 kb); SRF peak IDs; closest TSS gene name; distance (in bp) to the closest TSS. Columns 9–14. Effect of 30-minute TPA stimulation on each histone modification (0 or 1) as assessed by Deseq. Columns 15–20. Dependence on TCF of each histone modification (0 or 1), assessed by regression method. Columns 21–40. Normalized and averaged read counts for each of the 5 histone modifications in resting or TPA-stimulated (30') cells. Columns 41–52 Normalized and averaged H3S10ph, H3K9acS10ph and H3K4me3 read counts following 0, 5, 15 and 30 minutes TPA stimulation.

Table S3. PCR Primers, Related to Figures 4, 5, and 6 Summary of primers used for quantitative ChIP-PCR, and for qRT-PCR analysis of Egr1 and Fos pre-RNA and total RNA.

Table S4. siRNA Screening Results Summary, Related to Figures 6 and S6 The siRNA oligonucleotide pools and individual oligonucleotides used are shown. Pass denotes those which reduce TPA-induced level of Fos or Egr1 RNA or pre-RNA by at least 20% at p < 0.05 (Student’s t test). Data are shown in Figure S14.

Document S2. Article plus Supplemental Information
