# Supplementary material for: ERK-Induced Activation of TCF Family of SRF Cofactors Initiates a Chromatin Modification Cascade Associated with Transcription
Source: Mol Cell. 2017 Mar 16;65(6):1081–1095.e5. doi: 10.1016/j.molcel.2017.02.005 (PMC5364370; doi:10.1016/j.molcel.2017.02.005)
Supplement: Document S1. Figures S1–S7 [file mmc1.pdf]

Molecular Cell, Volume *65*

Supplemental Information

ERK-Induced Activation of TCF Family of SRF Cofactors Initiates a Chromatin Modification Cascade Associated with Transcription

Cyril Esnault, Francesco Gualdrini, Stuart Horswell, Gavin Kelly, Aengus Stewart, Phil East, Nik Matthews, and Richard Treisman

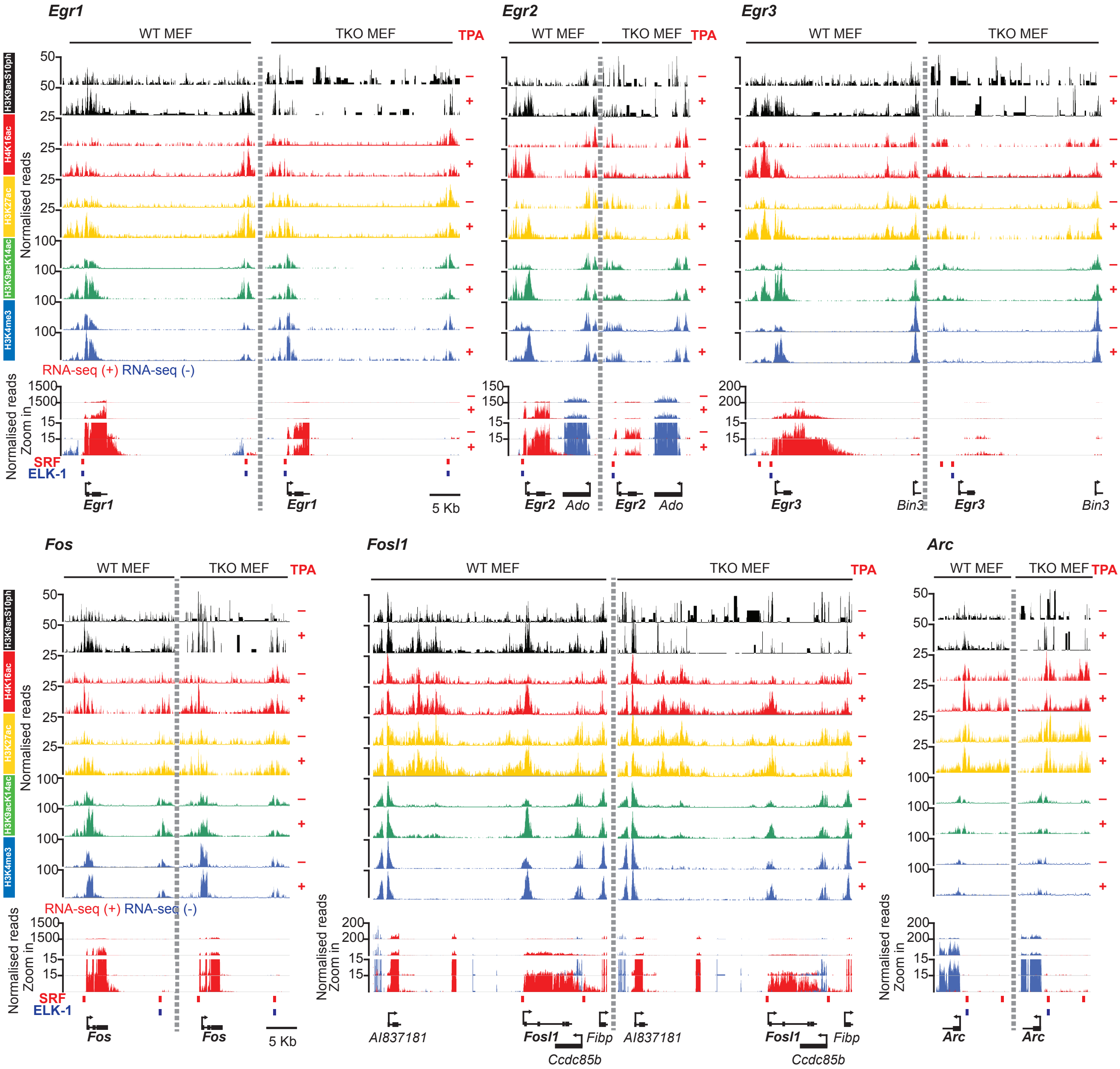
Egr1
WT MEF
TKO MEF
TPA
Egr2
Egr3
H3K9acS10ph
H4K16ac
H3K27ac
H3K9acK14ac
H3K4me3
Normalised reads
RNA-seq (+) RNA-seq (-)
Normalised reads
Zoom in
SRF
ELK-1
Ado
Bin3
5 Kb
Fos
Fosl1
Arc
AI837181
Fibp
Ccdc85b

Figure S1

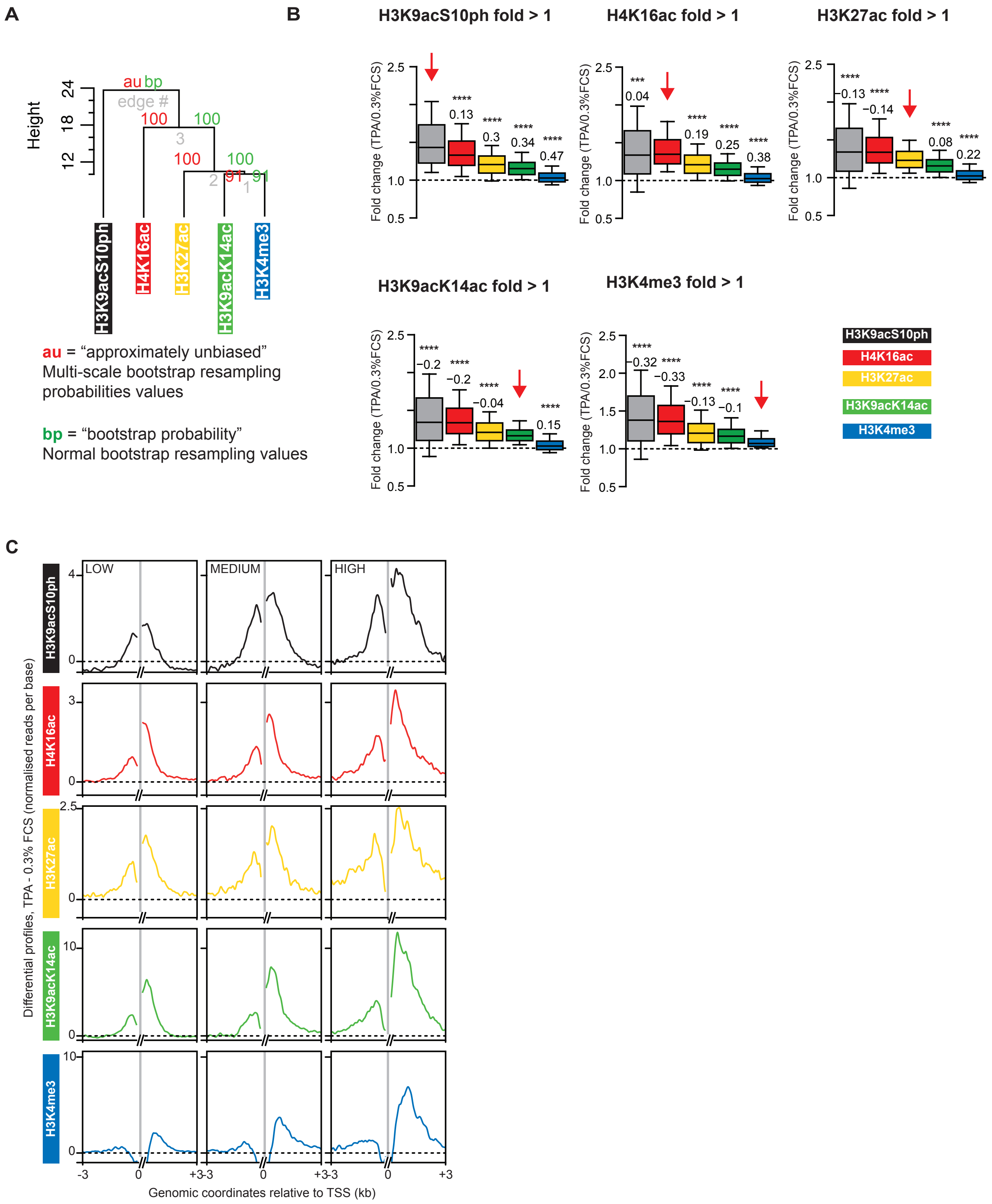

A
B
H3K9acS10ph fold > 1
H4K16ac fold > 1
H3K27ac fold > 1
H3K9acK14ac fold > 1
H3K4me3 fold > 1
Height
au bp
edge #
H3K9acS10ph
H4K16ac
H3K27ac
H3K9acK14ac
H3K4me3
au = "approximately unbiased"
Multi-scale bootstrap resampling probabilities values
bp = "bootstrap probability"
Normal bootstrap resampling values
Fold change (TPA/0.3%FCS)
C
LOW
MEDIUM
HIGH
Differential profiles, TPA - 0.3% FCS (normalised reads per base)
Genomic coordinates relative to TSS (kb)

Figure S2

A

Formulas	Coef.	Std. Er	Pr(>|t|)	R^2	F-statistic (p-val)
lm(RNA ~ H3K9acS10ph)	0.4	0.03	2.2E-48	0.07	< 2.2E-16
lm(RNA ~ H4K16ac)	0.8	0.04	9.2E-129	0.15	< 2.2E-16
lm(RNA ~ H3K27ac)	0.9	0.06	1.2E-93	0.11	< 2.2E-16
lm(RNA ~ H3K9acK14ac)	1.6	0.06	3.0E-192	0.23	< 2.2E-16
lm(RNA ~ H3K4me3)	2.4	0.07	3.2E-218	0.28	< 2.2E-16

B

Model 1 : stepAIC() from lm(RNA ~ 1) to lm(RNA ~ H3K9acS10ph+H4K16ac+H3K27ac+H3K9acK14ac+H3K4me3)

Model:lm(RNA ~ H3K4me3 + H3K9acK14ac + H4K16ac + H3K9acS10ph + H3K27ac)

Adjusted R-squared: 0.37
F-statistic: p-value: < 2.2e-16

Variables	Coef.	Std. Er	Pr(>|t|)	ΔRes. Dev
H3K4me3	1.70	0.09	2.2 E-74	373
H3K9acK14ac	0.48	0.08	1.7 E-09	72
H4K16ac	0.23	0.04	1.2 E-07	15
H3K9acS10ph	0.14	0.02	3.8 E-09	11
H3K27ac	0.27	0.06	2.7 E-06	7
(Intercept)	-0.04	0.02	ns	

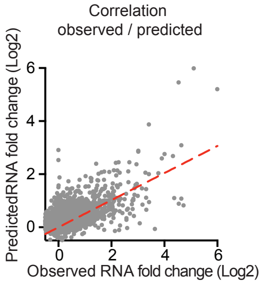

Slope = 0.5104 ± 0.01028
Spearman R = 0.50
P value (two-tailed) <0.0001 ****

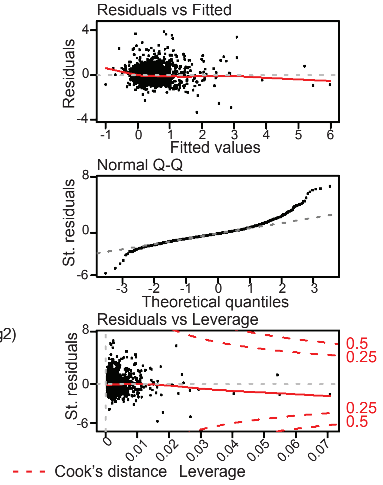

C

Model 2 (considering Synergy): stepAIC() from lm(RNA ~ 1) to lm(RNA ~ H3K9acS10ph * H4K16ac * H3K27ac * H3K9acK14ac * H3K4me3)

Model: lm(RNA ~ H3K4me3 + H3K9acK14ac + H4K16ac + H3K9acS10ph + H3K27ac + H4K16ac:H3K27ac + H3K4me3:H3K9acS10ph + H3K4me3 :H4K16ac + H3K9acK14ac:H3K9acS10ph + H3K4me3 : H3K9acK14ac + H4K16ac:H3K9acS10ph + H3K9acS10ph:H3K27ac)

Adjusted R-squared: 0.40
F-statistic: p-value: < 2.2e-16

Variables	Coef.	Std. Er	Pr(>|t|)	ΔRes. Dev
H3K4me3	1.81	0.12	7.0E-47	373.3
H3K9acK14ac	0.37	0.09	1.4E-05	72.1
H4K16ac	0.43	0.05	2.8E-16	15.5
H3K9acS10ph	0.12	0.04	1.3E-03	11.4
H3K27ac	0.49	0.07	9.0E-12	7.7
H4K16ac:H3K27ac	-0.30	0.09	1.1E-03	6.5
H3K4me3:H3K9acS10ph	0.57	0.14	6.7E-05	13.0
H3K4me3:H4K16ac	-1.32	0.25	1.3E-07	4.8
H3K9acK14ac:H3K9acS10ph	0.49	0.10	5.2E-07	4.3
H3K4me3:H3K9acK14ac	0.98	0.29	6.4E-04	4.6
H4K16ac:H3K9acS10ph	-0.18	0.07	8.1E-03	3.3
H3K9acS10ph:H3K27ac	-0.15	0.09	7.3E-02	1.1
(Intercept)	-0.11	0.03	5.3E-05	

anova(model.1,model.2): Sum of Sq = 37.46 Pr(>F) < 2.2e-16

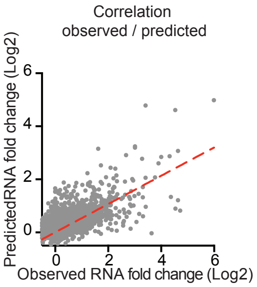

Slope = 0.5329 ± 0.01026
Spearman R = 0.52
P value (two-tailed) <0.0001 ****

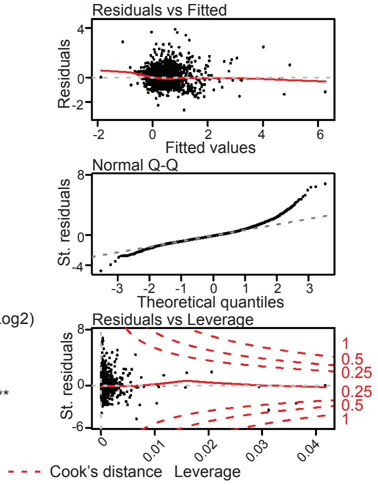

D

Model 2 (considering Synergy): stepAIC() from lm(RNA ~ 1) to lm(RNA ~ H3K9acS10ph * H4K16ac * H3K27ac * H3K9acK14ac * H3K4me3)

Model: lm(RNA ~ H3K4me3+ H3K9K14ac+ H3K9acS10ph+ H3K27ac+ H4K16ac+ H3K9acS10ph:H3K4me3 + H3K27ac:H3K9acK14ac + H3K9acS10ph:H3K27ac + H3K9acS10ph:H3K9acK14ac + H4K16ac:H3K27ac

Adjusted R-squared: 0.5
F-statistic: p-value: < 2.2e-16

Variables	Coef.	Std. Er	Pr(>|t|)	ΔRes. Dev
H3K4me3	1.8	0.24	3.5E-13	331
H3K9acK14ac	0.8	0.21	9.6E-05	31
H3K9acS10ph	0.2	0.08	2.4E-02	5
H3K9acS10ph:H3K4me3	0.8	0.27	2.9E-03	9
H3K27ac	0.8	0.15	4.7E-08	2
H3K27ac:H3K9K14ac	-0.7	0.33	2.8E-02	7
H3K9acK14ac:H3K27ac	-0.6	0.22	8.1E-03	2
H3K9acS10ph:H3K9acK14ac	0.6	0.25	2.2E-02	4
H4K16ac	0.3	0.11	2.4E-03	2
H4K16ac:H3K27ac	-0.6	0.27	3.4E-02	2
(Intercept)	-0.2	0.06	2.3E-04	

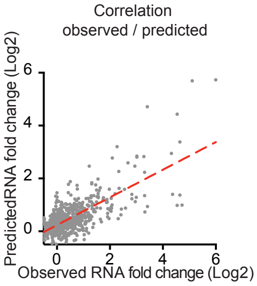

Slope = 0.5235 ± 0.01917
Spearman R = 0.55
P value (two-tailed) <0.0001 ****

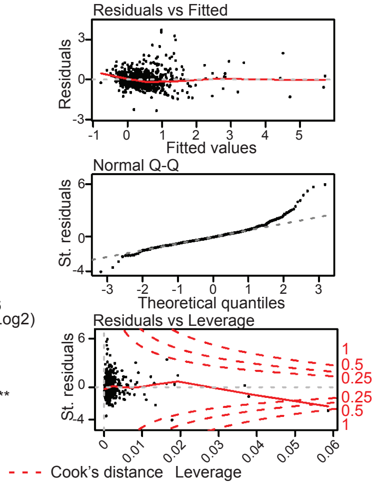

Figure S3

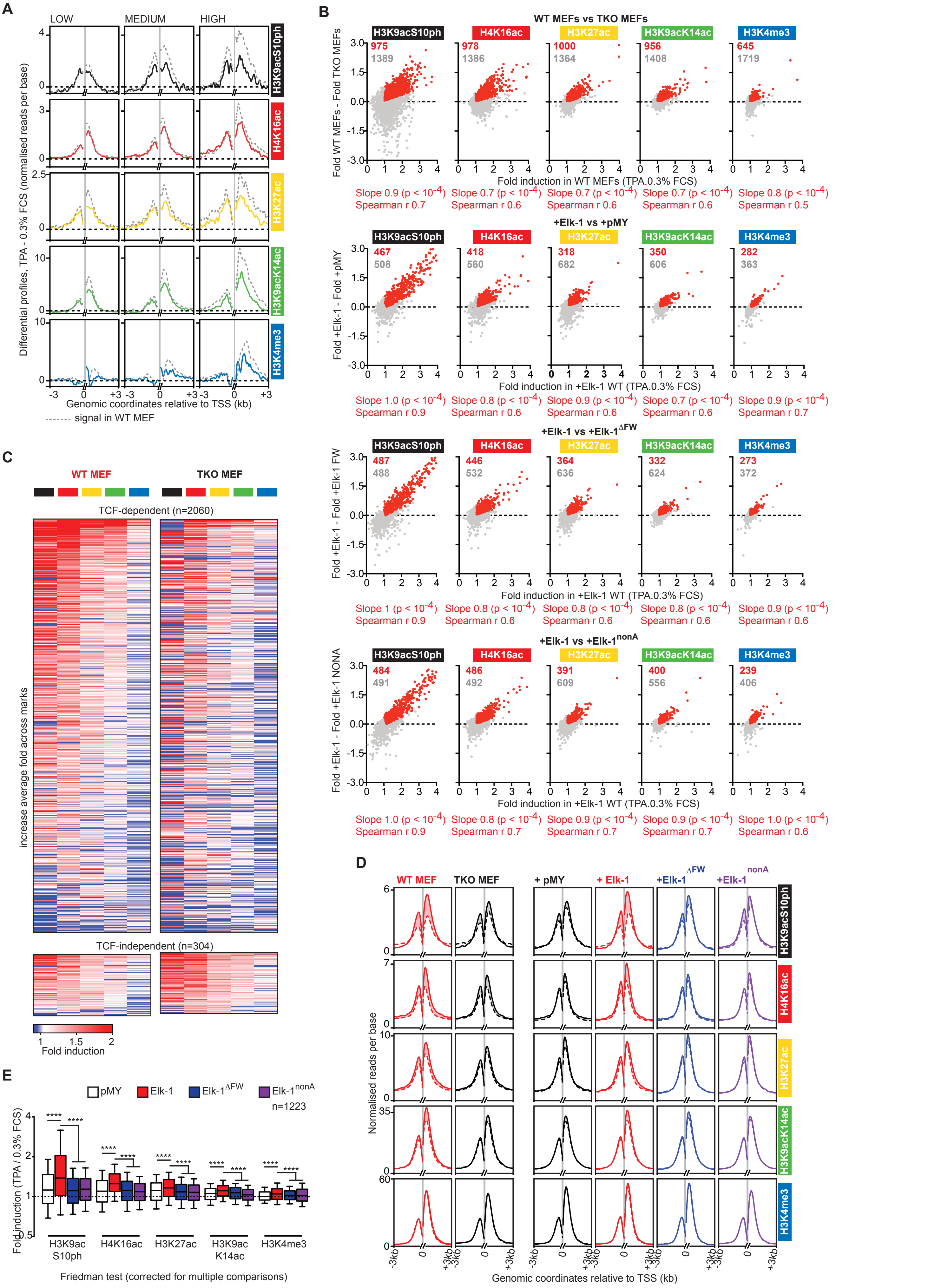
A
B
WT MEFs vs TKO MEFs
+Elk-1 vs +pMY
+Elk-1 vs +Elk-1ΔFW
+Elk-1 vs +Elk-1nonA
C
WT MEF
TKO MEF
TCF-dependent (n=2060)
TCF-independent (n=304)
Fold induction
D
E
Friedman test (corrected for multiple comparisons)

Figure S4

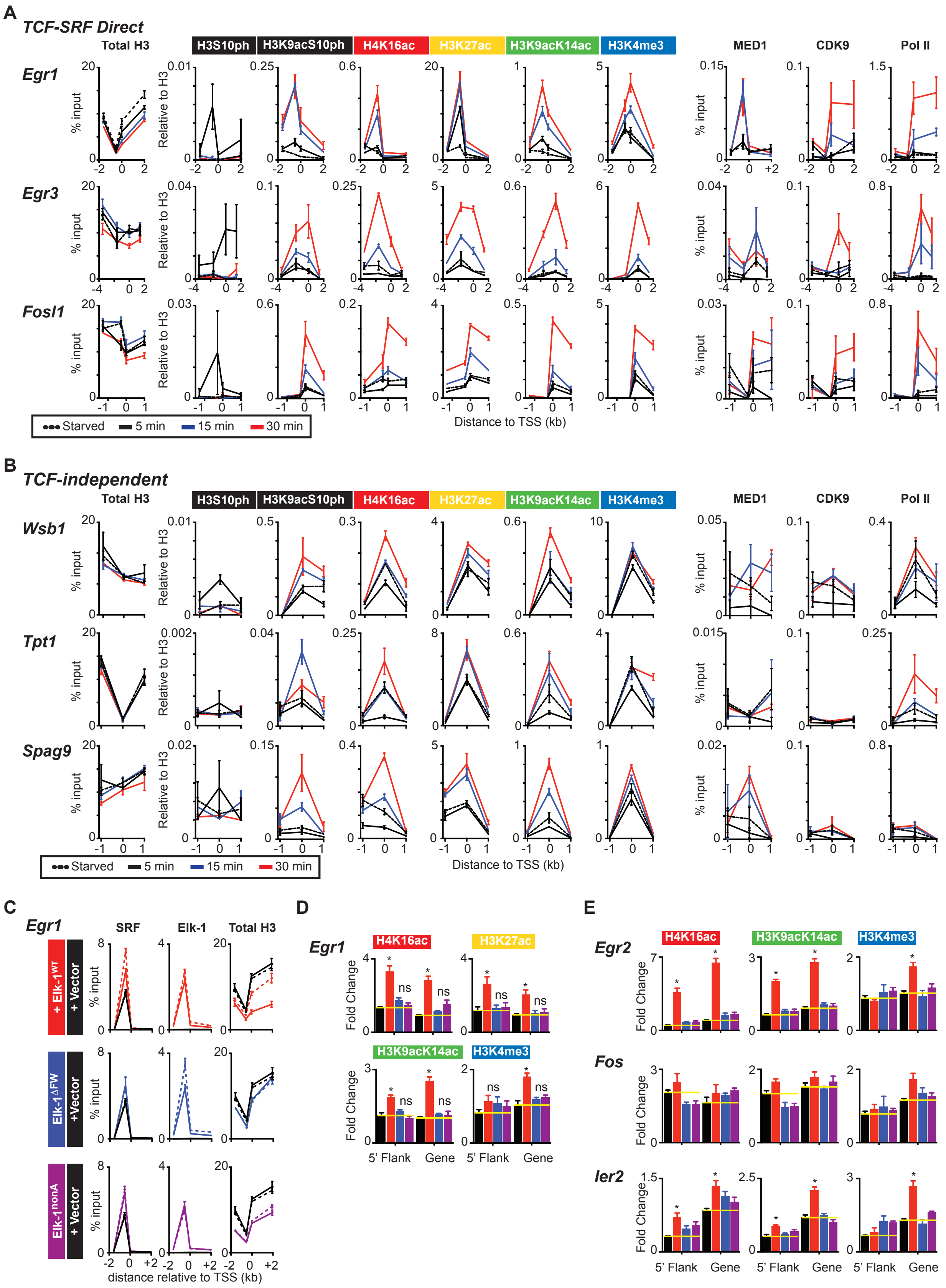
A
TCF-SRF Direct
Total H3
H3S10ph
H3K9acS10ph
H4K16ac
H3K27ac
H3K9acK14ac
H3K4me3
MED1
CDK9
Pol II
Egr1
Egr3
Fosl1
% input
Relative to H3
Starved
5 min
15 min
30 min
Distance to TSS (kb)
B
TCF-independent
Wsb1
Tpt1
Spag9
C
Egr1
SRF
Elk-1
Total H3
+ Vector
+ Elk-1WT
Elk-1ΔFW
Elk-1nonA
distance relative to TSS (kb)
D
Fold Change
5' Flank
Gene
ns
E
Egr2
Fos
Ier2

Figure S5

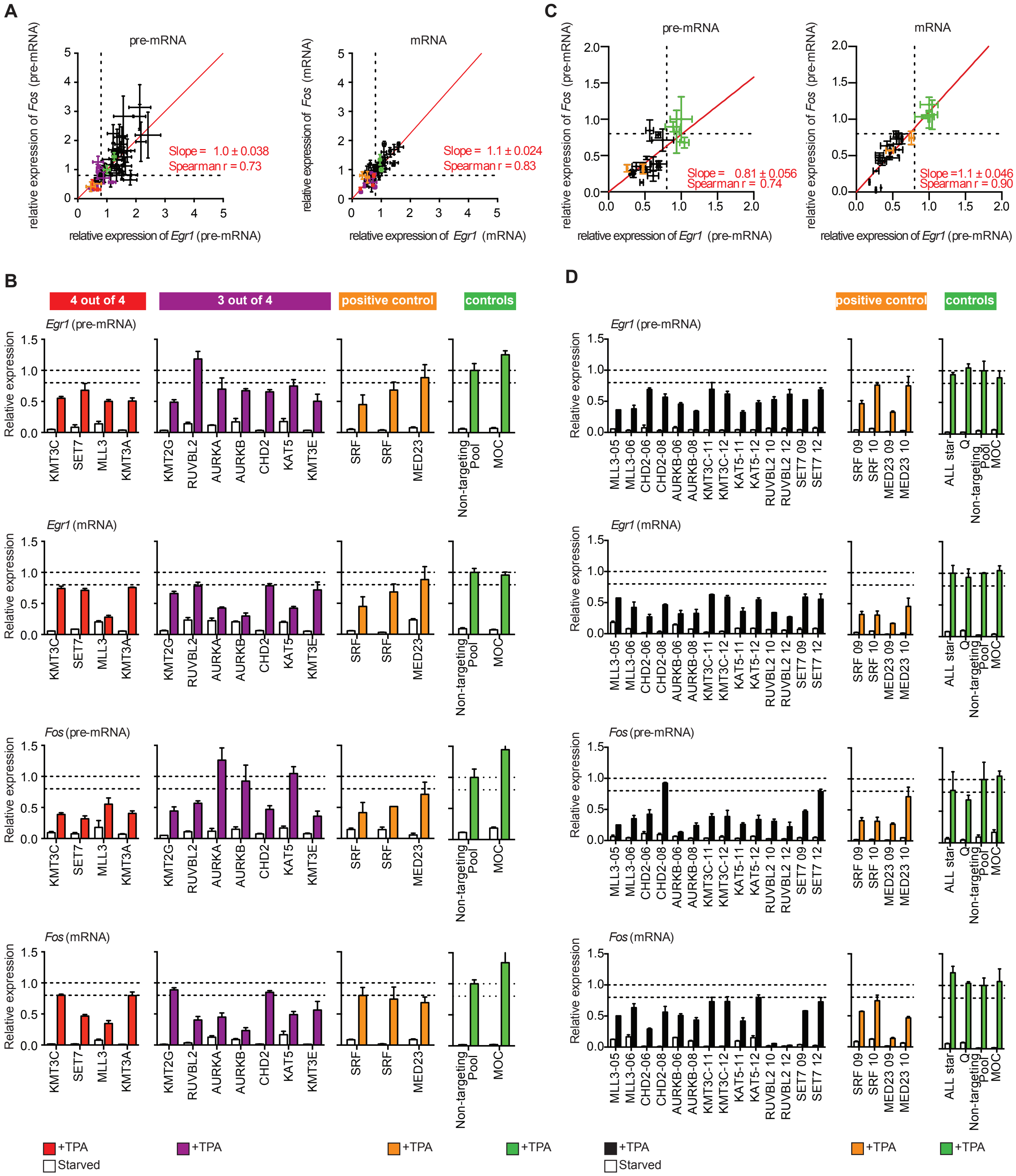
A
pre-mRNA
mRNA
relative expression of Fos (pre-mRNA)
relative expression of Fos (mRNA)
relative expression of Egr1 (pre-mRNA)
relative expression of Egr1 (mRNA)
Slope = 1.0 ± 0.038
Spearman r = 0.73
Slope = 1.1 ± 0.024
Spearman r = 0.83
C
Slope = 0.81 ± 0.056
Spearman r = 0.74
Slope =1.1 ± 0.046
Spearman r = 0.90
B
4 out of 4
3 out of 4
positive control
controls
Egr1 (pre-mRNA)
Egr1 (mRNA)
Fos (pre-mRNA)
Fos (mRNA)
Relative expression
KMT3C
SET7
MLL3
KMT3A
KMT2G
RUVBL2
AURKA
AURKB
CHD2
KAT5
KMT3E
SRF
MED23
Non-targeting Pool
MOC
D
MLL3-05
MLL3-06
CHD2-06
CHD2-08
AURKB-06
AURKB-08
KMT3C-11
KMT3C-12
KAT5-11
KAT5-12
RUVBL2 10
RUVBL2 12
SET7 09
SET7 12
SRF 09
SRF 10
MED23 09
MED23 10
ALL star
Q
+TPA
Starved

Figure S6

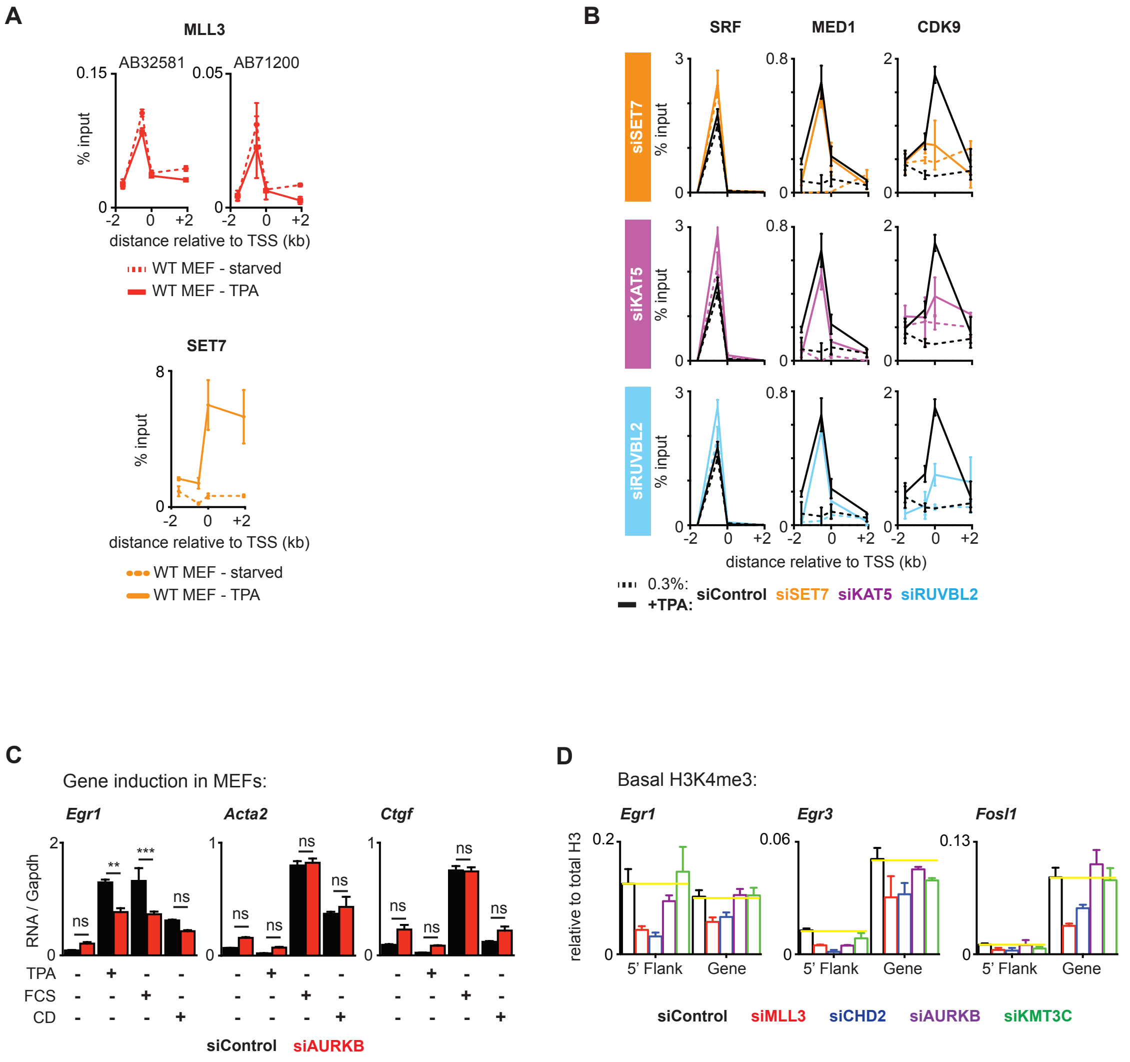
A
MLL3
AB32581
AB71200
% input
distance relative to TSS (kb)
WT MEF - starved
WT MEF - TPA
SET7
B
SRF
MED1
CDK9
siSET7
siKAT5
siRUVBL2
0.3%:
+TPA:
siControl siSET7 siKAT5 siRUVBL2
C
Gene induction in MEFs:
Egr1
Acta2
Ctgf
RNA / Gapdh
TPA
FCS
CD
siControl siAURKB
D
Basal H3K4me3:
Egr1
Egr3
Fosl1
relative to total H3
5' Flank
Gene
siControl siMLL3 siCHD2 siAURKB siKMT3C

Figure S7

SUPPLEMENTAL FIGURE LEGENDS

Figure S1 | (related to Figure 1) TPA-induced histone modifications at classical TCF-SRF target genes in wildtype and triply TCF-deficient MEFs.

IGB browser view of changes in histone modifications during a 30' TPA stimulation at *Egr1*, *Egr3*, *Fosl1*, *Egr2, Fos and Arc* in WT (left) and TKO MEFs (right). Black, H3K9acS10ph; red, H4K16ac; yellow H3K27ac; green H3K9acK14ac; blue H3K4me3. Below are shown RNA-seq prolifes in unstimulated and TPA-stimulated cells. Upper traces are scaled to show mRNA induction, lower traces are scaled to visualise eRNA and PROMPT transcripts. Genes are shown schematically below each plot; red flashes, SRF ChIP-seq peaks; blue flashes, Elk-1 ChIP-seq peaks (GEO: GSE75667; Gualdrini et al., 2016). Note that the histone modification profiles cannot resolve the FosL1 5' flanking from the intronic "enhancer" sequence, which binds AP1 components as part of the delayed-early response. *FosL1* S10 promoter and intronic sequences are reported to involve the Msk2/3 and Pim1 H3S10 kinases acting at early and late times respectively (Zippo et al., 2007). The TPA-induced changes in histone modifications at *FosL1* are are entirely dependent on TCF, which, as at *Egr1*, must therefore play the primary role in directing ERK signal to the gene.

Figure S2 | (related to Figure 1) Hierarchical clustering of TPA-induced histone modifications reflect their fold change similarity.

(A) Pvclust hierarchical clustering of the fold induction across the 5 modifications over multiscale bootstrap resampling (total 10000 iterations, method aiming to identify more accurate p-values) compared to simple bootstrapping method. The cluster *P*-value indicates how strong the cluster is supported by the data, and ranges between 0 and 100. "Approximately unbiased" probability ("AU", red) and the "bootstrap probability" ("BP", green) values are shown at each node. (B) Interdependency between histone mark fold changes. Panels show the distance between the increase in a particular histone modification and each of the other modifications. Box-plots show the overall unsmoothed distribution (horizontal line, median; box limits, 25th and 75th percentiles). The red arrow points to the histone modification considered as the independent variable, and numbers above each boxplot indicate the mean difference from it. Significance was determined by one-way ANOVA with Greenhouse-Geisser correction and Holm-Sidak's multiple comparison test: (***), $P<0.001$; (****), $P<0.0001$. (C) The 5 histone modifications for the 2364 TSS regions were grouped according to changes in H3K9acS10ph (Low, 1- to 1.5-fold, n=1093; Medium, 1.5- to 2-fold, n=598; High, ≥2-fold, n=216). Differential metaprofiles are shown as (TPA – 0.3% FCS) per base for each modification at the TSS regions displaying the greatest induction in H3K9acS10ph. See also Figure 1.

Figure S3 | (related to Figure 1) Regression analysis of the TPA-induced RNA synthesis as a function of the TPA-induced change in the 5 histone modifications.

(A) Linear regression of RNA synthesis (Log2 scaled) as function of fold changes in each histone modification (Log2 scaled). Regression was performed in R for each pair listed. The table summarises: the estimated coefficient of the regression (Coef.); the standard error associated with the estimated coefficient (Std. Er); the probability that the true coefficient is zero (Pr(>|t|); the adjusted R^2 or coefficient of determination, representing the proportion of the variance in the data that is explained by the model, corrected for the number of variables; the F-statistic (p-value) indicates the statistical significance of the prediction made by the model compared to random noise as predictor. (B) Multiple regression modelling with histone modifications considered independent variables. Stepwise model selection by using AIC (Akaike Information Criterion), to measure the relative quality of a statistical model for a given set of data, from lm(RNA ~ 1) to lm(RNA ~ H3K9acS10ph + H4K16ac + …). The table lists the independent variables in the order step AIC selected them, together with the estimated coefficient; the standard error associated with the coefficient, the probability that the estimated coefficient is zero; and the reduction in residuals deviation from the model (ΔRes.Dev.). The end product of stepAIC is reported at the bottom with the final equation, the adjusted R^2 and the F-statistic. The scatter plot shows the predicted RNA-fold changes estimated through the multiple regression plotted against the observed RNA changes. The red line indicates the linear fit between predicted and measured values; these are significantly correlated (Spearman R = 0.52),

indicating a good quantitative relationship between the levels of signal-induced histone modification and RNA production. Model validation graphs are shown below: “Residuals vs Fitted” to detect non-linearity, unequal error variances, and outliers; “Q-Q plot” to compare probability distributions between the two sets of data; “Residual vs Leverage” to value the influence of each observation on the regression coefficients (Cook’s distance statistic is a measure of the extent of change in model estimates when that particular observation is omitted). (C) Multiple regression modelling for all TPA-induced changes with histone modifications considered as interdependent variables. Stepwise model selection by using AIC, from lm(RNA ~ 1) to lm(RNA ~ H3K9acS10ph + H4K16ac + …). Data is organised as in panel (B). Anova test between the models in (B) and (C) shows that the latter significantly improve the model. (D) Multiple regression modelling for direct TCF direct target TSS (n=681) with histone modifications considered as interdependent variables, displayed as in (C).

Figure S4 | (related to Figure 2 and Figure 5) Characterisation of the role of TCFs in TPA-induced changes in histone modification.

(A) The 2364 TSS regions exhibiting TPA-induced changes in histone modifications in wildtype MEFs were grouped according to changes in H3K9acS10ph (Low, 1- to 1.5-fold, n=1093; Medium, 1.5- to 2-fold, n=598; High, ≥2-fold, n=216). Differential metaprofiles are shown as (TPA – 0.3% FCS) per base for each modification at the TSS regions displaying the greatest induction in H3K9acS10ph. Grey line covers 150bp on each side of the TSS excluded from the profiles, where low readcounts presumably reflect nucleosome depletion. Solid coloured lines, change in TKO MEFs; dotted grey, change in WT MEFs. See also Figure 1. (B) Estimation of the dependence of histone modifications on the TCFs, and the Elk-1 transcription activation domain (TAD). All TSS regions showing a significant change in histone modification upon TPA stimulation were considered (n = 2364; see Figure 1A). Changes were compared as described in Star Methods (Gualdrini et al., 2016). Data points included as significantly far from the y=0 axes are shown in red on each plot, with the slope and spearman r. The number of TSS regions deemed dependent on the condition under test is the union of the sets of red datapoints from each plot). First row, TCF-dependence of TPA-induced changes in each of the 5 histone modifications. Plots correlate the difference in fold-change upon TPA stimulation between WT and TKO MEFs with the fold-change in WT MEFs. 2060/2364 TSS regions exhibit TCF-dependence. Second row, TSS regions exhibiting Elk-1 dependent histone modifications. Comparison of TPA-induced changes in histone modifications in TKO cells re-expressing wildtype Elk-1 or pMY vector. 1223 TSS regions

show restoration of TPA-induced change in at least one histone modifications. Third and fourth rows, estimation of the number of TSS regions whose histone modifications depend on the Elk-1 TAD. TPA-induced changes in histone modifications in TKO cells re-expressing wildtype Elk-1 were compared with those in cells expressing Elk-1$^{\Delta FW}$ (FW motif deletion; third row) or Elk-1^{nonA} (unphosphorylatable TAD; fourth row). 1132 of 1223 Elk-1-dependent TSS regions showed dependence on the Elk-1 TAD. (C) Heatmap representations of the TPA-induced change in histone modifications at the 2060 TCF-dependent and 304 TCF-independent TSS regions in wildtype and TKO MEFs (H3K9acS10ph, black; H4K16ac, red; H3K27ac, yellow; H3K9acK14ac, green; H3K4me3, blue). (D) Metaprofiles of the 5 histone modifications at the 1233 TSS regions where in TKO cells expression of wildtype Elk-1 is sufficient to restore induction. Left panels, comparison of WT and TKO MEFs. For differential plot, see Figure 2D. Right panels, restoration of TPA-induced changes in histone modification is TKO cells expressing pMY vector alone (black) or expressing either wildtype Elk-1 or the Elk-1^{nonA} or Elk-1$^{\Delta FW}$ TAD mutants. (E) Boxplot representation of the average fold induction of each histone mark in TKO MEFs reconstituted with empty pMY vector (white), Elk-1 (red), Elk-1$^{\Delta FW}$ (blue) or Elk-1^{nonA} (purple) at the 1223 TSS regions that exhibit TCF-dependent histone changes (see Figure 2D, 2E). Middle line, median; top and bottom edges, 75th and 25th percentiles; horizontal bars, 90th and 10th percentiles. Statistical significance by Friedman test with Dunn's multiple comparison test per set of histone modifications. (****), P<0.0001; (**), P<0.01.

Figure S5 | (related to Figures 4, 5) Signal-induced histone modifications

(A-B) Time course of histone modification and transcriptional machinery recruitment at direct SRF-TCF targets (i.e. *Egr1*, *Egr3* and *Fosl1*) and at TPA-induced TCF independent targets (*Wsb1*, *Tpt1* and *Spag9*). Using PCR probes as in Figure 4A. The time course was performed at t=0 (dotted); 5 min (black); 15 min (blue) and 30 min (red) following TPA stimulation. Total H3, Med1, Cdk9 and PolII signal is shown as relative to the input material while H3S10ph, H3K9acS10ph, H4K16ac, H3K27ac, H3K9acK14ac and H3K4me3 are shown as relative to total H3. Data are means ± SEM, n=4. (C) Quantitative ChIP of SRF, Elk-1 and total H3 in wildtype and TKO MEFs. Data are means ± SEM, n=3. (D) Quantitative ChIP of histone modifications at *Egr1* in TKO MEF cells reconstituted with Elk-1 (red), Elk-1$^{\Delta FW}$ (blue), Elk-1^{nonA} (purple) or with pMY vector (black). Data are means ± SEM, n=3. (E) Quantitative qPCR-ChIP of histone modifications at *Egr1, Egr2, Fos or Ier2* in TKO MEF cells reconstituted with Elk-1 derivatives as in (D).

Figure S6 | (related to Figure 6) siRNA screen for chromatin regulators required for TPA induced *Egr1* and *Fos* transcription.

siRNA oligonucleotide pools against 50 catalytic subunits of chromatin regulatory complexes (Table S2) were screened for their ability to inhibit TPA-induced in *Egr1* and *Fos* transcription, each assessed at pre-RNA and mRNA level using qRT-PCR. Positive controls (orange) were siRNAs targeting SRF or MED23; negative controls (green) were mock-transfection, Dharmacon ON-TARGETplus Non-targeting Pool siRNA, AllStar siRNA (Qiagen) or Q (Negative Control siRNA; Qiagen). (A) The siRNAs have similar effects on *Egr1* and *Fos* transcription. Plots show correlation between the relative expression levels of *Egr1* and *Fos* pre-mRNAs (left) or mRNAs (right) after TPA stimulation of siRNA-transfected cells. A decrease of at least 20% in TPA-induced level (at $p<0.05$ by Student's t test), similar to that seen on SRF depletion, was considered significant. (B) Hits identified in the primary screen as impairing 4 (red) or 3 (purple) of the transcriptional 4 readouts are shown, together with positive (orange) and negative (green) control. Dotted lines indicate normal induction (100%), and that observed upon SRF-depletion (80%). (C) Deconvoluted siRNAs from the hits identified in (B), show similar effects on relative expression levels of *Egr1* and *Fos* pre-mRNAs (left) or mRNAs (right) after TPA stimulation of siRNA-transfected cells. (D) Inhibition of *Egr1* and *Fos* induction by individual deconvoluted siRNAs targeting MLL3, CHD2, AURKB, KMT3C, KAT5, RUVBL2 and SET7. Labelling as in (B). All data are means ± SEM, n=3.

Figure S7 | (related to Figure 6) Functional analysis of chromatin regulator functions at *Egr1*.

(A) Quantitative ChIP analysis of MLL3 and SET7 binding at *Egr1*. Probes as in Figure 4A. (B) Quantitative ChIP analysis of SRF, MED1, and CDK9 binding at *Egr1* following depletion of SET7 (orange), KAT5 (lilac), RUVBL2 (blue) or scrambled oligonucleotide control (black), analysed as in Figure 4A. (C) siAURKB does not affect *Acta2* or *Ctgf* activation. qRT-PCR analysis of MEFs treated with AURKB or control siRNAs, treated with TPA, 15% FCS or 2μM Cytochalasin D (CD) for 30 minutes as indicated.(D) Quantitative ChIP of H3K4me3 at *Egr1*, *Egr3* and *Fosl1* in unstimulated cells following depletion of MLL3 (red), CHD2 (blue), AURKB (purple) or KMT3C (green). Scrambled oligonucleotide control (black). H3K4me3 signal was normalised to H3. All data are means ± SEM, n=3. See also Figure 6C.

SUPPLEMENTAL TABLE LEGENDS

Table S1 | ChIP-seq runs (related to Figures 1, 2, 3)

Summary of the ChIP-seq runs, listing Run name, Cell source, Genotype and/or transfectant, Antibody data, and scaling factors. Data is available at GSE75002.

Table S2 | Summary of histone modification ChIP-seq data at TSS regions and DNAse I HS (related to Figures 1, 2, 3, 4, S3, S4).

(A) TPA-induced changes occurring in TSS regions

Columns 1-6. Gene description: Official gene symbol; transcript ID; Genomic coordinates (Chromosome, Strand, Start and Stop).

Columns 7-10. Relationship between each TSS and the 2577 SRF ChIP-seq peaks, including closest SRF peak coordinate; distance to the closest peak; whether the TSS is linked to SRF by Hi-C; Direct targets for TCF-SRF histone regulation defined those TSS exhibiting TPA-induced histone modification located 10kb of an SRF peak and/or linked to one by Hi-C (n=817).

Columns 11-16. Summary of the RNA-seq data (Gualdrini et al., 2016) including TPA-induction (0 or 1); Direct TCF-SRF targets, as assessed by comparison of RNA-seq and integrated SRF ChIP-seq / Hi-C data (n=763); total and intronic RNA read counts in WT MEF before and after 30' TPA stimulation.

Columns 17-22. Effect of 30 minute TPA stimulation on each histone modification. TPA-induction (0 or 1).

Columns s 23-28. Dependence of each induced histone modification on TCF (0 or 1) obtained by comparison of wildtype and TKO MEFs using regression method.

Columns 29-34. Restoration of induced histone modifications (0 or 1) by expression of wildtype Elk-1 in TKO MEFs, assessed by regression method.

Columns 35-40. Restoration of induced histone modifications (0 or 1) by expression of Elk-1 activation-domain mutants in TKO MEFs, assessed by regression method.

Columns 41-100. Normalised and averaged histone modification read counts in resting or TPA-stimulated (30') cells, for each of the 5 antibodies..

Columns 101-108. Normalised and averaged read counts for each of the 5 histone modifications in resting or TPA-stimulated (30') cells.

(B) Changes occurring at the DNAse I HS sites.

Column 1-4. Coordinates for all DNAse I HS sites which show TPA-induced change in at least one histone modification (n=2404).

Columns 5-8. Relation of each DNAse I HS site to SRF ChiP-seq peaks and closest TSS. Number of SRF peaks coincident with the DNAse I HS region (peak ± 2kb) ; SRF peak IDs; closest TSS gene name; distance (in bp) to the closest TSS.

Columns 9-14. Effect of 30-minute TPA stimulation on each histone modification (0 or 1) as assessed by Deseq.

Columns 15-20. Dependence on TCF of each histone modification (0 or 1), assessed by regression method.

Columns 21-40. Normalised and averaged read counts for each of the 5 histone modifications in resting or TPA-stimulated (30') cells.

Columns 41-52 Normalised and averaged H3S10ph, H3K9acS10ph and H3K4me3 read counts following 0, 5, 15 and 30 minutes TPA stimulation.

Table S3 | PCR primers (related to Figures 4, 5, 6)

Summary of primers used for quantitative ChIP-PCR, and for qRT-PCR analysis of Egr1 and Fos pre-RNA and total RNA.

Table S4 | siRNA screening results summary (related to Figures 6, S6)

The siRNA oligonucleotide pools and individual oligonucleotides used are shown. Pass denotes those which reduce TPA-induced level of Fos or Egr1 RNA or pre-RNA by at least 20% at $p<0.05$ (Student's t-test). Data is shown in Figure S14.
